# Human resident liver myeloid cells protect against metabolic stress in obesity

**DOI:** 10.1038/s42255-023-00834-7

**Published:** 2023-07-06

**Authors:** Emelie Barreby, Benedikt Strunz, Sebastian Nock, Léa Naudet, Joanne X. Shen, Helene Johansson, Isabella Sönnerborg, Junjie Ma, Egon Urgard, Laura J. Pallett, Yizhou Hu, Achilleas Fardellas, Valerio Azzimato, Ana Vankova, Laura Levi, Cecilia Morgantini, Mala K. Maini, Per Stål, Stephan P. Rosshart, Jonathan M. Coquet, Greg Nowak, Erik Näslund, Volker M. Lauschke, Ewa Ellis, Niklas K. Björkström, Ping Chen, Myriam Aouadi

**Affiliations:** 1 Center for Infectious Medicine (CIM), Department of Medicine Huddinge, Karolinska Institutet, Karolinska University Hospital, Stockholm, Sweden; 2 Department of Physiology and Pharmacology, Karolinska Institutet, Solna, Sweden; 3 Division of Transplantation Surgery, Department of Clinical Science, Intervention and Technology, Karolinska Institutet (CLINTEC), Huddinge, Sweden; 4 Department of Microbiology, Tumor and Cell Biology (MTC), Karolinska Institutet, Stockholm, Sweden; 5 Division of Infection and Immunity, Institute of Immunity and Transplantation, University College London, London, United Kingdom; 6 Division of Molecular Neurobiology, Department of Medical Biochemistry and Biophysics, Karolinska Institutet, Stockholm, Sweden; 7 Division of Gastroenterology, Department of Medicine, Huddinge, Karolinska Institutet, Stockholm, Sweden; 8 Department of Microbiome Research, Friedrich-Alexander-University Erlangen-Nürnberg, Erlangen, Germany; 9 Department of Medicine II, Medical Center, Faculty of Medicine, University of Freiburg, Freiburg im Breisgau, Germany; 10 Division of Surgery, Department of Clinical Sciences, Danderyd Hospital, Karolinska Institutet, Stockholm, Sweden; 11 Dr Margarete Fischer-Bosch Institute of Clinical Pharmacology, Stuttgart, Germany; 12 University of Tuebingen, Tuebingen, Germany; 13 Present Address: BioPharmaceuticals R&D, Clinical Pharmacology and Safety Sciences, Translational Hepatic Safety, AstraZeneca, Gothenburg, Sweden; 14 Present Address: Cardio Metabolic Unit, Department of Medicine Huddinge, Karolinska Institutet, Karolinska University Hospital, Stockholm, Sweden; 15 Present Address: Division of Clinical Chemistry, Department of Laboratory Medicine, Karolinska Institutet, Stockholm, Sweden

**Keywords:** Obesity, Monocytes and macrophages

## Abstract

Although multiple populations of macrophages have been described in the human liver, their function and turnover in patients with obesity at high risk of developing non-alcoholic fatty liver disease (NAFLD) and cirrhosis are currently unknown. Herein, we identify a specific human population of resident liver myeloid cells that protects against the metabolic impairment associated with obesity. By studying the turnover of liver myeloid cells in individuals undergoing liver transplantation, we find that liver myeloid cell turnover differs between humans and mice. Using single-cell techniques and flow cytometry, we determine that the proportion of the protective resident liver myeloid cells, denoted liver myeloid cells 2 (LM2), decreases during obesity. Functional validation approaches using human 2D and 3D cultures reveal that the presence of LM2 ameliorates the oxidative stress associated with obese conditions. Our study indicates that resident myeloid cells could be a therapeutic target to decrease the oxidative stress associated with NAFLD.

## Abstract (web summary)

Barreby et al. identify a distinct subpopulation of human resident liver myeloid cells that expresses factors that act in a protective fashion against oxidative stress associated with NAFLD.

## Main

NAFLD is a condition in which hepatic fat accumulation leads to tissue damage. NAFLD can develop into non-alcoholic steatohepatitis (NASH), an aggressive form of fatty liver disease, which is projected to become the leading cause of liver-related morbidity and mortality within 20 years and the most common indication for liver transplantation in the next few years^1^. Despite its high prevalence of 3–5% in the general population and potential life-threatening effects^2^, no approved treatments for NASH are currently available. This highlights the unmet need to improve our understanding of the underlying pathogenic and pathological mechanisms.

Although patients with obesity and NAFLD are at the greatest risk of developing NASH, surprisingly little is known about the function and turnover of distinct liver macrophage subsets during these early stages of the disease. Although informative, single-cell RNA sequencing (scRNA-seq) studies have characterized macrophage diversity in patients with obesity and cirrhosis with different etiologies^3,4^, but the function and turnover of these liver macrophage populations remain unknown. Due to the lack of human fate-mapping models, previous studies have examined human macrophage expression profiles that characterize liver macrophage populations in the context of the mouse macrophage ontogeny literature^3–8^. However, how conserved murine liver macrophage diversity is in human individuals, with respect to their turnover and functions, remains unknown. We combined single-cell approaches using Smart-seq2 scRNA-seq to obtain full-transcript coverage and high resolution of cell populations, multicolor flow cytometry and in vitro two-dimensional (2D) and three-dimensional (3D) cultures to define the phenotypes, origin and functions of liver myeloid (LM) cell subpopulations during metabolic disease. We identified a distinct subpopulation of resident LMs expressing factors protective against the development of obesity-associated oxidative stress. Considering the dramatic damaging consequences of oxidative stress in the liver, our study implicates new treatment options for liver disease.

## Results

### Characterization of human LM cells during the development of NAFLD

**Fig. 1:**
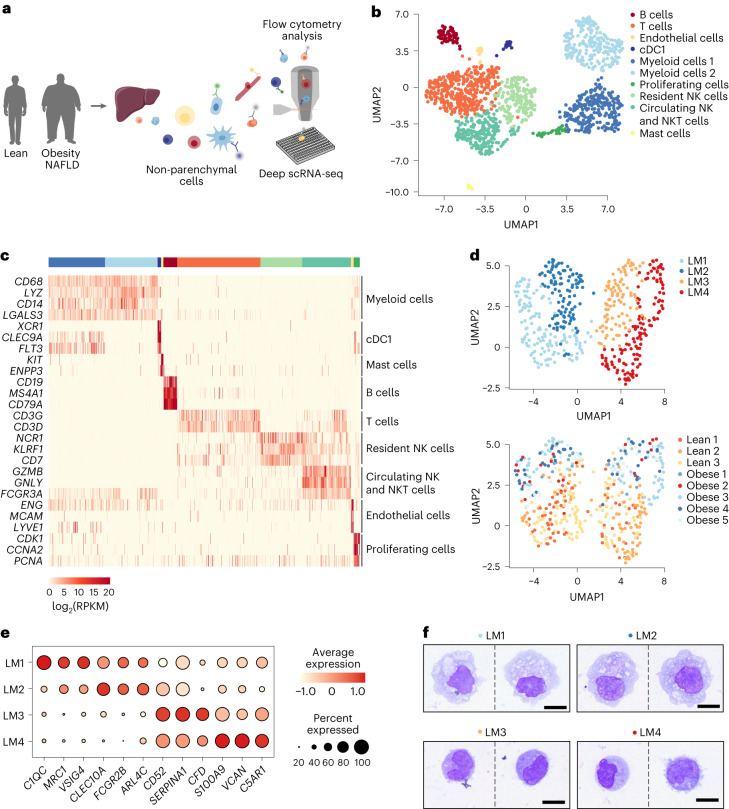
Identification of distinct LM cell populations in livers of lean humans and humans with obesity. **a**, Experimental outline: human NPCs are isolated from livers of lean patients and patients with obesity. NPCs are then single-cell sorted using an antibody panel with 11 markers to record the expression of cell-surface proteins for individual cells, followed by single-cell transcriptomic profiling. **b**, Uniform manifold approximation and projection (UMAP) visualization of NPCs from lean (*n* = 3) individuals and individuals with obesity (*n* = 5); colors indicate cell cluster. Each symbol represents a single cell. **c**, Gene expression (log_2_(RPKM)) of markers for each cell type. RPKM, reads per kilobase of exon model per million mapped reads. **d**, UMAP visualization of LM cells from lean individuals (*n* = 3) and individuals with obesity (*n* = 5) colored by subpopulations (top) and colored by individual donors (bottom). **e**, Dot plot of marker genes significantly differentially expressed by each individual myeloid cell subpopulation. Color intensity indicates expression level, and dot size indicates gene expression frequency (percentage of cells expressing the gene). **f**, Representative images of cytospins stained with Wright-Giemsa of respective LM cell population, sorted from one lean individual. Scale bar, 10 µm. Illustrations in **a** were partly created using components adapted from Servier Medical Art, provided by Servier, licensed under a Creative Commons Attribution 3.0 unported license. Source data

**Extended Data Fig. 1:**
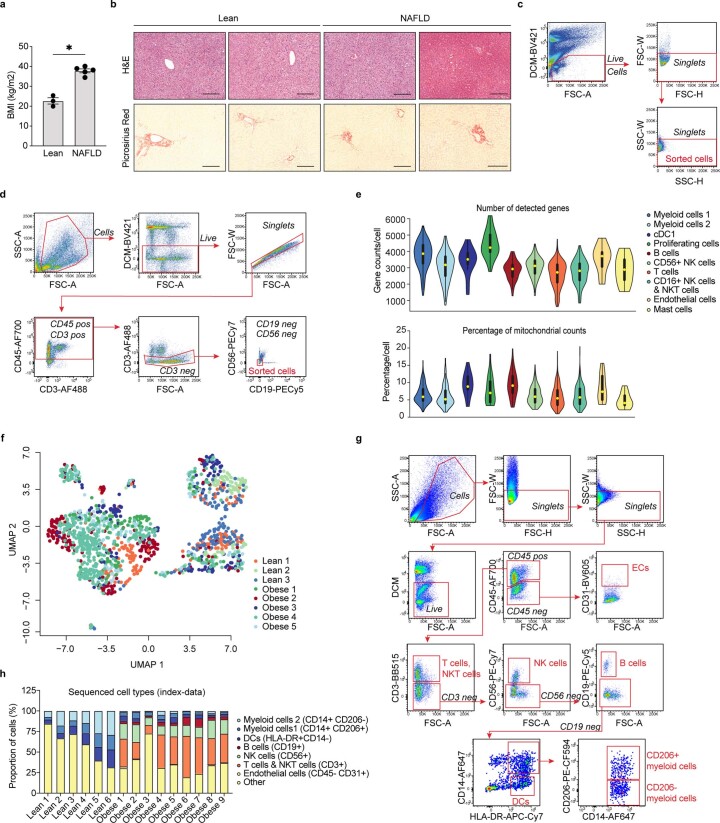
Characterisation of human liver non-parenchymal cells. a, Average BMI of sequenced individuals (n=3 lean and n=5 with obesity; P=0.0357). b, Representative images of H&E and picrosirius red staining of livers from lean individuals and individuals with obesity and NAFLD. Scale bar, 200µm. Images are representative of 5 individuals per condition (cohort 1). c, Gating scheme for sorting of NPCs from patients with obesity. Sorted cells were gated as live, single cells. d, Gating scheme for sorting of myeloid cells from lean individuals. Sorted cells were gated as live, single cells, CD45^+^CD3^-^CD19^-^CD56^-^ cells. e, Violin plots of the number of detected genes (top) and the percentage of mitochondrial genes (bottom) across non-parenchymal cells from lean (n=3) and NAFLD (n=5) livers. Yellow dot indicates the median, bold line indicates the interquartile range and thin line displays the 1.5x interquartile range. f, UMAP visualization of single NPCs coloured by individual donors. g, Gating scheme for flow cytometric analysis of all sequenced NPCs (from the FACS-index data) gated using fluorescence minus one (FMO) controls and h, proportion of cell types from all sequenced plates from livers of lean individuals (n=6 plates) compared to individuals with obesity (n=9 plates). Data are presented as mean ± s.e.m. P values were calculated two-tailed Mann-Whitney test. *p < 0.05, **p < 0.01, ***p < 0.001, and ****p < 0.0001. ns, not significant. BMI, body mass index; NAFLD, Non-alcoholic fatty liver disease; H&E, haematoxylin and eosin; DCM, dead cell marker. Source data

**Extended Data Fig. 2:**
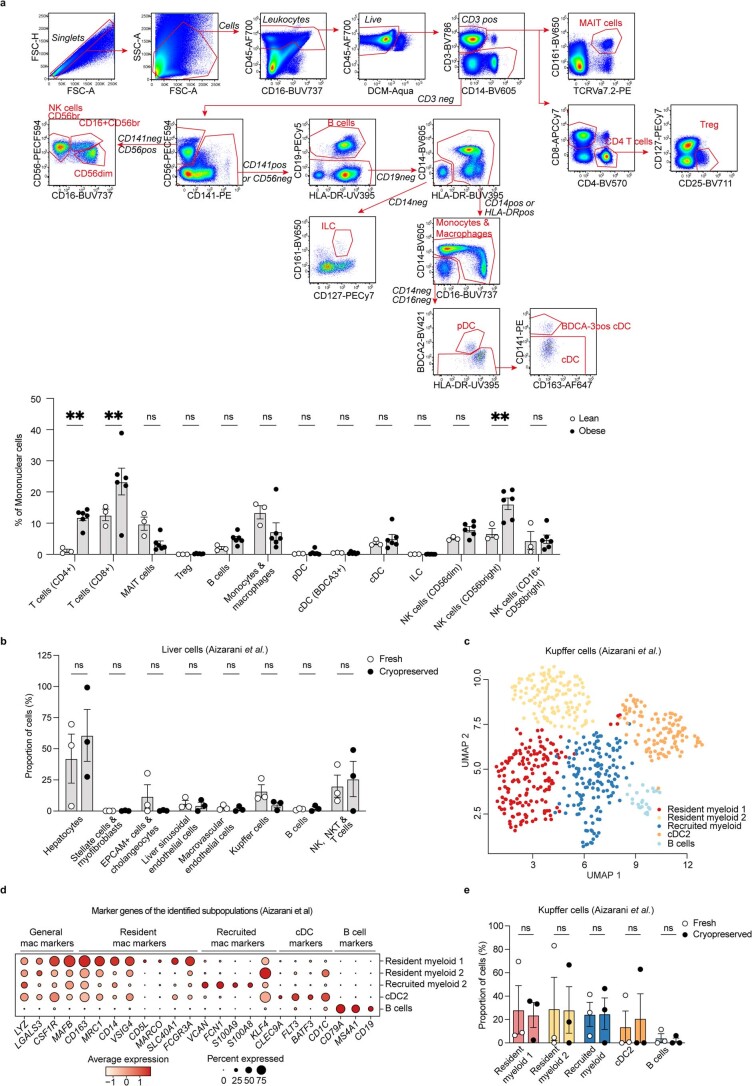
Characterisation of human liver non-parenchymal cells. a, Top: Gating scheme for flow cytometric analysis of human NPCs from lean individuals and individuals with obesity. Black regular text indicates markers used for each gating, black italic text indicates the selected population and red text the final subset. Several clean up steps have been implemented that are not displayed here, such as clean-up of staining artefacts, removal of cells expressing CD14/CD19/CD4 for final NK cell population, and removal of TCRVa7.2/CD4 for final ILC population. Bottom: Proportion of immune cell subsets among all live CD45^+^ cells after flow cytometry analysis. Results are from lean perfused liver samples (n=3) and obese non-perfused liver samples (n=6) (CD4 T cells lean vs obese P=0.0010, CD8 T cells lean vs obese P=0.0012 and CD56bright NK cells lean vs obese P=0.0067). b, Proportion of sequenced human liver cells from fresh (n=3) and cryopreserved (n=3) samples by scRNA-seq from the previously published data set (GSE124395) by Aizarani et al. c, UMAP visualization of the Kupffer cell cluster annotated by Aizarani et al.; colours indicate subpopulations from reanalysis. Each symbol represents a single cell. d, Dot plot of marker genes for each subpopulation. Colour intensity indicates expression level and dot size indicates gene expression frequency (percentage of cells expressing the gene) by Aizarani et al. e, Proportion of fresh and cryopreserved cells for each subpopulation in the Kupffer cell cluster (n=3 liver samples per group) from Aizarani et al. Data are presented as mean ± s.e.m. P values were calculated by two-way ANOVA with adjustment for multiple comparisons. **p < 0.01. ILC, innate lymphoid cells; Treg, regulatory T cells; Classic. mono, classical monocyte; Interm. mono, intermediate monocyte; nonclassic. mono, nonclassical monocyte; conv. mDC, conventional mDC; CD56br, CD16^-^CD56^bright^ NK cells; CD16^+^CD56^br^, CD16^+^CD56^bright^ NK cells; CD56^dim^, CD56^dim^ NK cells. Mac, macrophages. Source data

To characterize LM cell heterogeneity and plasticity during early development of obesity-associated NAFLD, we performed full-length scRNA-seq of non-parenchymal cells (NPCs) isolated from the livers of lean individuals (body mass index (BMI) ≤ 25) and individuals with obesity (BMI > 35) (Fig. 1a and Extended Data Fig. 1a). All individuals with obesity presented with steatosis, as indicated by the hepatic steatosis index (HSI > 36), without any histological signs of fibrosis (Table 1 and Extended Data Fig. 1b). Viable NPCs from the cohort with obesity were unbiasedly sorted and sequenced using a modified version of the Smart-seq2 protocol adapted for liver cells, in order to obtain deep sequencing data with full-transcript coverage (Extended Data Fig. 1c)^9^. Prior to sorting, NPCs were stained with an antibody panel covering immune and non-immune cell markers to enable simultaneous characterization of the surface protein signature of each sorted cell. Myeloid cells were enriched in the lean cohort by sorting CD45^+^CD3^−^CD19^−^CD56^−^ cells (Extended Data Fig. 1d). Transcriptomic analysis of cells from both lean individuals and individuals with obesity identified clusters of myeloid cells, conventional dendritic cells (cDCs), mast cells, B cells, T cells, natural killer (NK) cells, natural killer T (NKT) cells, endothelial cells and a cluster of proliferating cells (Fig. 1b,c, Extended Data Fig. 1e and Supplementary Table 1). The majority of all identified clusters contained cells from each individual without any apparent batch effect or population bias due to cryopreservation prior to sequencing, as evident from both scRNA-seq and fluorescence-activated cell sorting (FACS) analyses (Extended Data Figs. 1f–h and 2a–e).

**Table 1:** Clinical characteristics of patients included in the study: cohort 1

Clinical parameter	Lean (*n* = 12)	Obesity (*n* = 13)
Age, years (mean, range)	62 (26–83)	40 (28–56)
Sex, female/male (*n*)	6/6	13/0
BMI, kg m^−^^2^, (mean, range)	23 (20–25)	38 (35–41)
Diabetes (*n*)	NA	3
HSI (mean, range)	NA	52 (44–60)
HbA1c, mmol mol^−1^ (mean, range)	NA	39 (27–57)
HOMA-IR	NA	6.1 (2.0–10.4)
Fasting insulin, mIU l^−1^ (mean, range)	NA	23 (11–75)
Fasting glucose, mmol l^−1^ (mean, range)	NA	6.2 (4.6–8.3)
Creatinine, µmol l^−1^ (mean, range)	NA	65 (45–89)
ALT, µkat l^−1^ (mean, range)	NA	0.6 (0.3–1.4)
AST, µkat l^−1^ (mean, range)	NA	0.4 (0.2–0.8)
GGT, µkat l^−1^ (mean, range)	NA	1.2 (0.2–3.5)
Total cholesterol, mmol l^−1^ (mean, range)	NA	5.1 (3.7–6.4)
LDL cholesterol, mmol l^−1^ (mean, range)	NA	3.1 (1.7–4.3)
HDL cholesterol, mmol l^−1^ (mean, range)	NA	1.2 (1.0–1.5)
Triglycerides, mmol mol^−1^ (mean, range)	NA	2.0 (0.7–3.4)

HbA1c, hemoglobin A1c; IU, international units; ALT, alanine aminotransferase; AST, aspartate aminotransferase; GGT, gamma glutamyl transferase; LDL, low-density lipoprotein; HDL, high-density lipoprotein; NA, not applicable.

**Extended Data Fig. 3:**
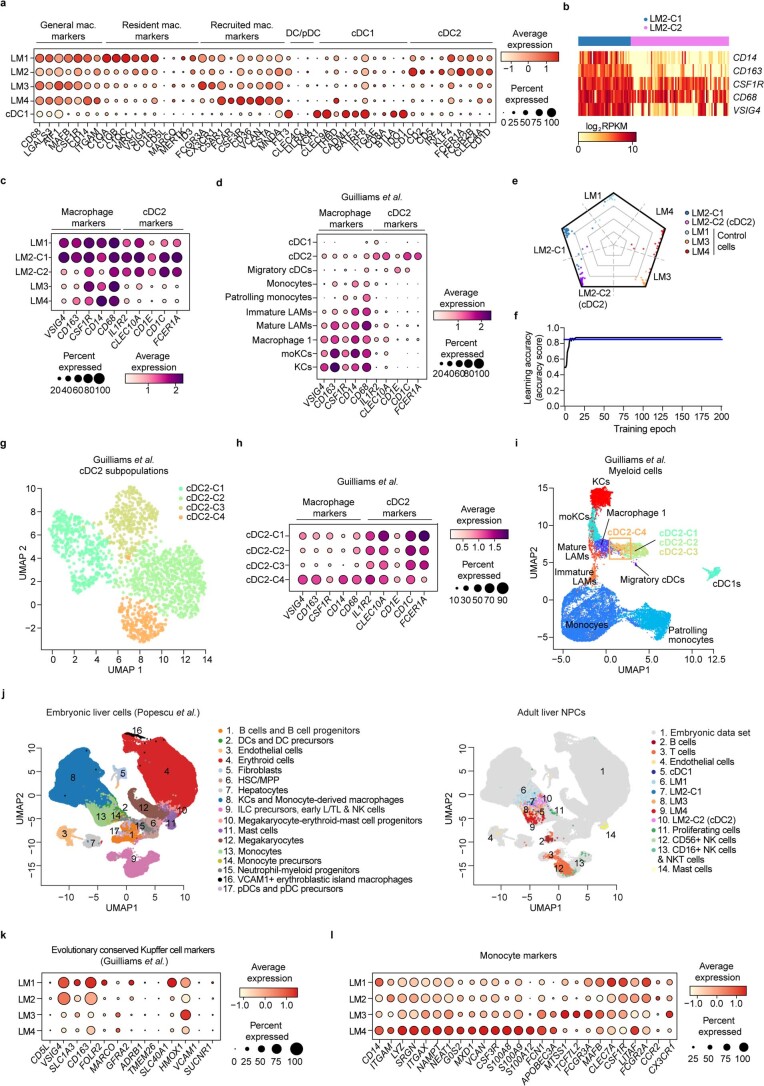
Characterisation and annotation of human liver myeloid cells. a, Dot plot of gene expression (log_2_ RPKM) of macrophage and dendritic cell markers. Colour intensity indicates expression level and dot size indicates gene expression frequency (percentage of cells expressing the gene). b, Heatmap of gene expression (log_2_ RPKM) of macrophage markers in the LM2 and cDC2 subclusters. c, Dot plot of gene expression of macrophage and cDC2 marker in our human myeloid cell data and d, in a published data set (GSE192742) of human myeloid cells by Guilliams et al. e, Radar plot visualization of the assigned cell-type score by the neural network classifier for each individual cell in the LM2/cDC2 subclusters and control cells; colours indicate cell cluster. f, Accuracy of the neural-network classifier in learning 261 single cells of LM2, cDC2 and the control cell types (LM1, LM3 and LM4) visualized as a learning curve. g, Subcluster analysis of cDC2 cells from the published data set of human myeloid cells by Guilliams et al. and h, dot plot of macrophage and cDC2 markers for each subcluster. i, UMAP visualisation of the cDC2 subclusters from g in relation to other myeloid populations. Orange rectangle indicates position of cluster cDC2-4. j, Integration of the human NPC data with a published data set (E-MTAB-7407) of human embryonic liver cells by Popescu et al. k, Dot plot of gene expression of previously published evolutionary conserved marker genes of Kupffer cells in mice and humans by Guilliams et al. l, Dot plot of gene expression (log_2_ RPKM) of monocyte markers. Data are presented as mean ± s.e.m. P values were calculated by two-way ANOVA with adjustment for multiple comparisons. RPKM, Reads per kilobase of exon model per million mapped reads; pDC, plasmacytoid dendritic cells; cDC1, type 1 dendritic cells; cDC2, type 2 dendritic cells; KC, Kupffer cells; moKCs, monocyte-derived KCs; LAMs, lipid-associated macrophages; HSC/MPP, hematopoietic stem cells and multipotent progenitors; early L/TL, early lymphoid/T lymphocyte.

Further analysis of the myeloid cell clusters revealed the presence of four transcriptionally distinct CD68^+^ liver myeloid cell subpopulations (LM1–LM4) in both lean individuals and individuals with obesity (Fig. 1d,e and Supplementary Table 2). The LM1 subset was characterized by the expression of markers previously associated with liver resident macrophages (Kupffer cells (KCs)) (*VSIG4*, *MRC1*, *C1QC*, *MERTK, SIGLEC1* and high levels of *CD163*)^3,5,7^ (Fig. 1e, Extended Data Fig. 3a and Supplementary Table 2). The LM2 cluster also expressed some of these markers (*VSIG4*, *CD163* and *MRC1*) along with genes such as *CLEC10A*, *FCGR2B* and high levels of *ARLC4C*. In addition to expressing general monocyte and macrophage-specific genes (for example, *CSF1R* and *MAFB*), the LM2 cells expressed genes shared with dendritic cells (for example, *CD1C*, *FLT3* and *FCER1A*) (Extended Data Fig. 3a and Supplementary Table 2). This was not surprising, as certain dendritic cell subsets are transcriptionally very similar to liver macrophages^4^. Further analysis revealed that the LM2 cluster contained a group of cells (LM2-C2) with low expression of macrophage-specific genes (*CD68*, *CD14, CD163*, *CSF1R* and *VSIG4*) while expressing markers of type 2 cDCs (cDC2) as previously observed^4^, suggesting their identity as cDC2s (Extended Data Fig. 3b–d). Only the remaining cells (LM2-C1) that expressed these macrophage markers at high levels (*CD68*, *CD14, CD163*, *CSF1R* and *VSIG4*) were annotated as LM2 and were used for subsequent analyses. The distinct identities of LM2 (LM2-C1) and LM2-C2 (cDC2-like cells) were further confirmed in silico by a neural network classifier (learning accuracy > 87%) using LM1, LM3 and LM4 cells as control (Extended Data Fig. 3e, f). Interestingly, subcluster analysis of a previously published single-cell dataset of human liver myeloid cells also identified a subpopulation of cDC2 cells (cDC2-C4) expressing both macrophage and cDC2 markers, similar to our LM2 cells (Extended Data Fig. 3g–i)^4^. The LM2 cells (LM2-C1) thus represent a unique population of myeloid cells co-expressing macrophage and dendritic cell (DC) markers. It would therefore be interesting to dissect the origin of this population during development in future studies.

**Extended Data Fig. 4:**
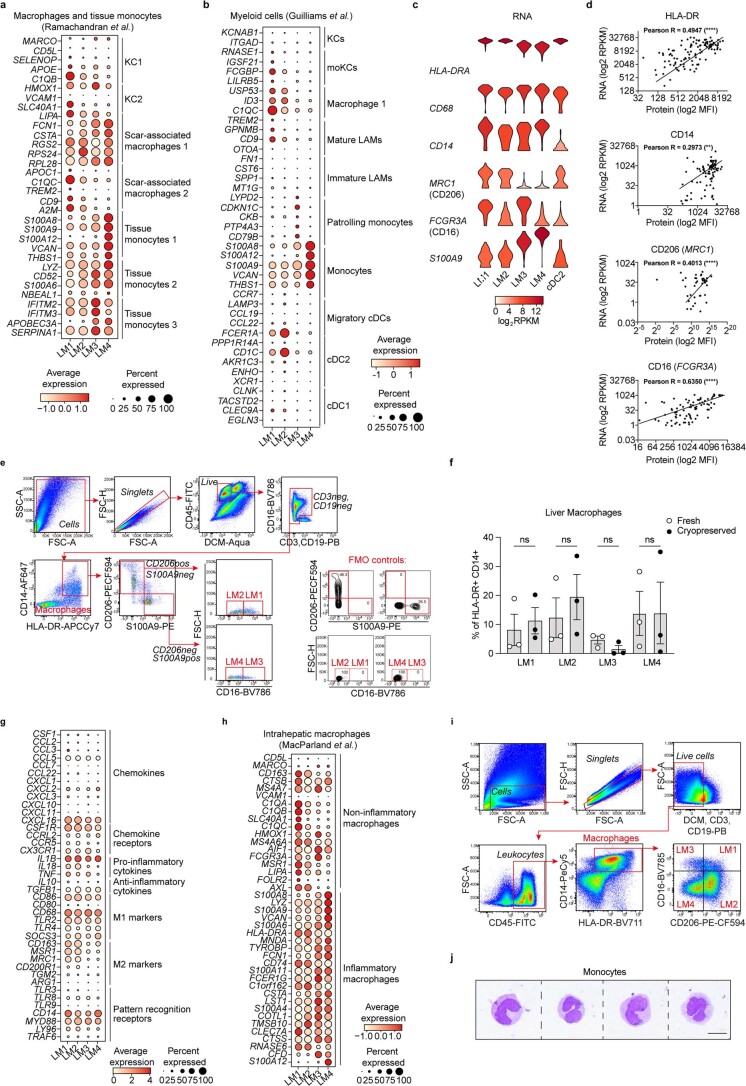
Characterisation and annotation of human liver myeloid cells. a, Dot plot of gene expression (log_2_ RPKM) of previously published liver macrophage markers (top DEG for each cluster) by Ramachandran et al. Colour intensity indicates expression level and dot size indicates gene expression frequency (percentage of cells expressing the gene). b, Dot plot of previously published myeloid cell markers (top DEG for each cluster) by Guilliams et al. c, Violin plot of gene expression distribution (log_2_ RPKM; colours indicate mean expression) of signature markers used to define each LM cell population by flow cytometry analysis or sorting. Myeloid cells were defined as HLA-DR^+^CD68^+^ and LM1 was defined as CD14^+^CD206^+^CD16^+^S100A9^low^, LM2 was defined as CD14^+^CD206^+^CD16^-^S100A9^low^, LM3 was defined as CD14^+^CD206^-^CD16^+^S100A9^high^, LM4 was defined as CD14^+^CD206^-^CD16^-^S100A9^high^ and cDC2 was defined as CD14^-^CD206^+^CD16^-^S100A9^low^. d, Correlation between RNA (log_2_ RPKM) and protein expression (log_2_ MFI; from indexed data) of selected signature markers on single LMs (HLA-DR P<0.0001, CD4 P=0.0011, CD206 P<0.0001, CD16 P<0.0001). Data are from 5 individuals with obesity. e, Gating scheme for flow cytometry analysis of human NPCs (representative gating plots are from a lean individual) gated using FMO controls and f, proportion of human LM subset in lean individuals and individuals with obesity before (fresh, n=3) and after cryopreservation (n=3). g, Dot plot of gene expression (log_2_ RPKM) of chemokines, chemokine-receptors, cytokines, M1 and M2 markers, and pattern recognition receptors in LM subsets. h, Dot plot of gene expression (log_2_ RPKM) of previously published liver macrophage marker genes by MacParland et al. i, Gating scheme for sorting of each individual LM subset (LM1-LM4) from human NPCs by FACS, gated using FMO controls. Representative gating plots are from a lean individual. j, Representative images of cytospins stained with Wright-Giemsa of isolated monocytes from peripheral blood mononuclear cells from one individual. Scale bar, 10µm. Data are presented as mean ± s.e.m. P values were calculated by two-way ANOVA with adjustment for multiple comparisons (f) or by two-tailed Pearson correlation (confidence interval: 95%). *p < 0.05. RPKM, Reads per kilobase of exon model per million mapped reads; MFI; mean fluorescence intensity; ns, not significant; KC, Kupffer cells; moKCs, monocyte-derived KCs; LAMs, lipid-associated macrophages. Source data

Integration of our data with a published dataset of human embryonic liver cells further demonstrated similarities between our LM1 and LM2 populations and embryonic tissue-resident macrophages^10^ (embryonic KCs and monocyte-derived macrophages) (Extended Data Fig. 3j). LM1 cells also displayed a phenotype described as ‘evolutionary conserved KCs,’ a set of marker genes of long-lived liver-resident KCs identified in mice and also recorded to be expressed by human resident macrophages (Extended Data Fig. 3k)^4^. Additionally, our LM3 and LM4 subpopulations overlapped with the embryonic population of monocytes (Extended Data Fig. 3j). LM3 and LM4 populations also exhibited similarities with adult liver monocytes or recently recruited monocyte-derived liver macrophages from other published datasets^3,4^, suggesting a monocyte-like phenotype (Extended Data Figs. 3l and 4a, b). Using the signatures defined through the scRNA-seq and flow cytometric recordings of cell-surface proteins for the LM cell subpopulations (Supplementary Table 3 and Extended Data Fig. 4c, d), we also analyzed each population using flow cytometry before and after cryopreservation. We failed to observe any significant effect of cryopreservation on LM cell population proportions (Extended Data Fig. 4e,f). Although gene ontology analysis revealed enrichment of terms such as ‘immune activation’ in all four subsets, they could not be clearly distinguished by their inflammatory phenotypes (Extended Data Fig. 4g and Supplementary Table 4). Only the LM4 cells expressed markers indicative of a modest pro-inflammatory phenotype (Supplementary Tables 2 and 3). LM4 cells were also transcriptionally similar to recruited macrophages, previously described as being pro-inflammatory despite their modest expression of inflammatory genes (Extended Data Fig. 4g, h)^5^. The lack of inflammatory activation of recruited compared with resident LMs was not surprising considering the general immunosuppressive environment of the liver^6,11,12^. Moreover, sorted LM1–LM4 cells analyzed using Cytospin exhibited a morphology comparable to previously reported human KCs (LM1 and LM2) and monocyte-derived macrophages (LM3 and LM4)^10^, but distinct to monocytes from the circulation, confirming their tissue monocyte or macrophage identity (Fig. 1f and Extended Data Fig. 4i, j). Taken together, these results suggest a different origin of LM1 and LM2 (resident) compared with LM3 and LM4 (monocyte-derived) cells. Furthermore, our data reveal that human LMs may be distinguished by their ontogeny rather than by their inflammatory status.

**Fig. 2:**
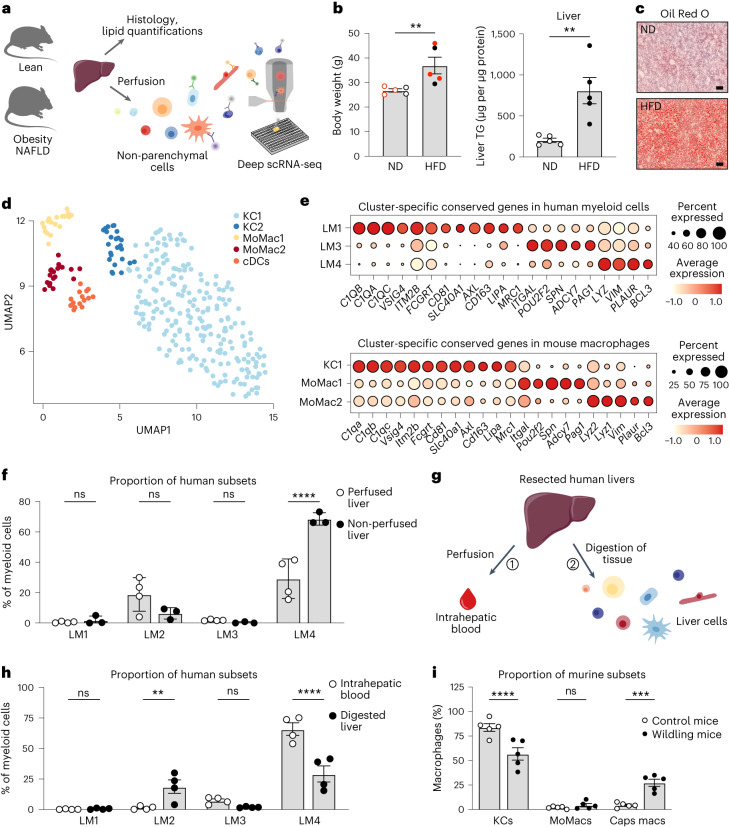
Human–mouse conservation of the LM cell populations. **a**, Experimental outline: livers from lean and obese mice on a high-fat diet for 9 weeks were either collected for histology and lipid quantifications or perfused to isolate murine NPCs. NPCs were single-cell sorted using an antibody panel with ten markers to record the expression of cell-surface proteins for individual cells and used for single-cell transcriptomic profiling. **b**, Body weight (*n* = 5 per group; *P* = 0.0079) and lipid (triglyceride, TG) content in murine livers (*n* = 5 per group; *P* = 0.0079). Red indicates mice used for scRNA-seq. ND, normal diet; HFD, high-fat diet. **c**, Representative images of lipid staining with Oil Red O of murine livers (*n* = 3 per group). Scale bar, 50 µm. **d**, UMAP visualization of liver myeloid cells from lean (*n* = 3) and obese mice (*n* = 3); colors indicate cell cluster. Each symbol represents a single cell. **e**, Dot plot of conserved genes between humans (top) and mice (bottom) specifically expressed by each macrophage cluster. Color intensity indicates expression level, and dot size indicates gene expression frequency (percentage of cells expressing the gene). **f**, Proportion of human LM subsets among all living CD45^+^CD68^+^HLA-DR^+^ myeloid cells in perfused (*n* = 4) and non-perfused livers (*n* = 3) (LM4 perfused versus non-perfused, *P* < 0.0001). ns, not significant. **g**, Experimental outline: (1) resected livers were perfused to flush out the intrahepatic blood; (2) livers were then digested and cells from the intrahepatic blood and the digested liver tissues were compared using flow cytometry. **h**, Proportion of LM subsets among all living CD45^+^CD68^+^HLA-DR^+^ myeloid cells in intrahepatic blood and digested liver tissue from the same donors (*n* = 4) (LM2 blood versus liver, *P* = 0.0084; LM4 blood versus liver, *P* < 0.0001). **i**, Proportion of murine LM cells in livers from wildling (*n* = 5) and control (*n* = 5) mice (KCs control versus wildling, *P* < 0.0001; Caps macs control versus wildling, *P* = 0.0003). Caps macs, capsular macrophages. Data are presented as mean ± s.e.m. *P* values were calculated by two-tailed Mann–Whitney *U*-test (**b**) or two-way ANOVA with adjustment for multiple comparisons (**f**, **h**, **i**). **P < 0.01; ***P < 0.001; ****P < 0.0001. Illustrations in **a** and **g** were partly created using components adapted from Servier Medical Art, provided by Servier, licensed under a Creative Commons Attribution 3.0 unported license. Source data

**Extended Data Fig. 5:**
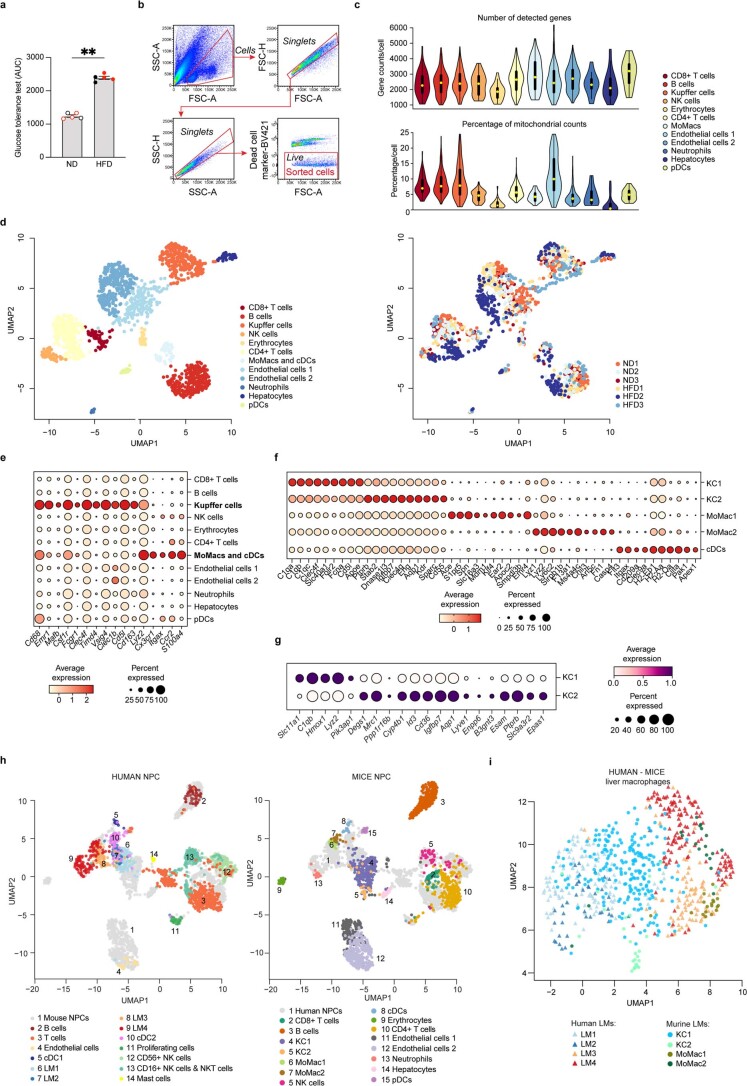
Characterisation and annotation of murine liver macrophages. a, Glucose tolerance in murine livers (n=5 per condition; P=0.0079), red colour indicates mice used for scRNA-seq. b, Gating scheme for sorting of NPCs from lean mice (n=3) and mice with obesity (n=3) for scRNA-seq. Sorted cells were gated as live, single cells. c, Violin plots of the number of detected genes (left) and the percentage of mitochondrial genes (right) across non-parenchymal cells from lean (n=3) and NAFLD (n=3) murine livers. Yellow dot indicates the median, bold line indicates the interquartile range and thin line displays the 1.5x interquartile range. d, UMAP visualization of NPCs from lean mice (n=3) and mice with obesity (n=3); colours indicate cell cluster (left) and individual mouse donors (right). Each symbol represents a single cell. e, Dot plot of gene expression of previously published markers of Kupffer cells and monocyte-derived macrophages. Colour intensity indicates expression level and dot size indicates gene expression frequency (percentage of cells expressing the gene). f, Dot plot of differentially expressed genes for each myeloid cell subpopulation. g, Dot plot of differentially expressed genes for the Kupffer cell 1 (KC1) and Kupffer cell 2 (KC2) subpopulations. h, Single cell integration of human and mouse scRNA-seq data of NPCs and i, LM cells. Data are presented as mean ± s.e.m. P values were calculated by two-tailed Mann-Whitney test. **p < 0.01. AUC, area under the curve; ND, normal diet; HFD, high fat diet; MoMacs, monocyte-derived macrophages.

**Extended Data Fig. 6:**
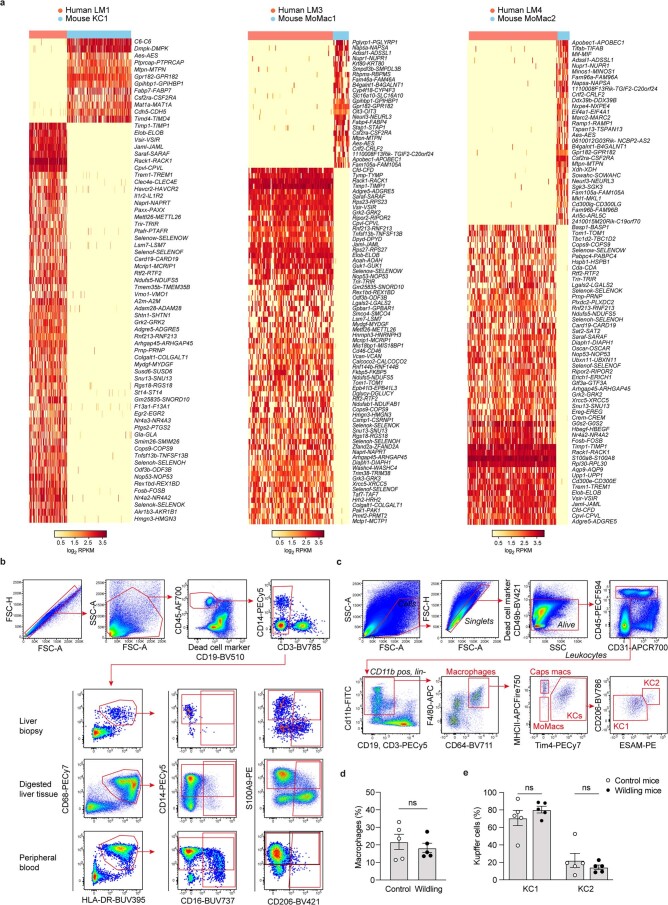
Human-mouse conservation of the LM cell populations. a, Heatmaps of genes specifically expressed (log_2_ RPKM) by humans or by mice for each matched LM cell subpopulation present in both mice and humans. b, Gating scheme for flow cytometry analysis of human livers and intrahepatic blood, where cells were gated amongst all live CD45^+^CD68^+^HLA-DR^+^ myeloid cells. LM1 was defined as CD14^+^CD16^+^CD206^+^S100A9^-^, LM2 as CD14^+^CD16^-^CD206^+^S100A9^-^, LM3 as CD14^+^CD16^+^CD206^-^S100A9^+^, and LM4 as CD14^+^CD16^-^CD206^-^S100A9^+^. c, Gating scheme for flow cytometry analysis of macrophage subpopulations in murine livers of wildling and specific pathogen-free (SPF) control mice, gated using FMO controls. d, Proportion of murine liver macrophages in wildling (n=5) and SPF control (n=5) livers. Liver macrophages are gated as live CD45^+^ lineage^-^(CD49b, CD3, CD19) CD11b^+^ F4/80^+^ CD64^+^. e, Proportion of murine KC subsets in wildling (n=5) and SPF control (n=5) livers. KC1 is gated as live CD45^+^ lineage^-^(CD49b, CD3, CD19) CD11b^+^ F4/80^+^ CD64^+^ Tim4^+^ CD206^-^ ESAM^-^ and KC2 is gated as CD45^+^ lineage^-^(CD49b, CD3, CD19) CD11b^+^ F4/80^+^ CD64^+^ Tim4^+^ CD206^+^ ESAM^+^. Data are presented as mean ± s.e.m. P values were calculated by two-tailed Mann-Whitney test (d) or two-way ANOVA with adjustment for multiple comparisons (e). *p < 0.05. RPKM, Reads per kilobase of exon model per million mapped reads; LM, liver myeloid cells; KC, Kupffer cell; MoMacs, monocyte-derived macrophages; Caps macs, capsular macrophages. Source data

Next, we investigated the conservation of LM cell diversity in humans and mice by performing scRNA-seq of NPCs from lean mice and mice with obesity presenting with liver steatosis and insulin resistance (Fig. 2a–c, Extended Data Fig. 5a–d and Supplementary Table 5). Transcriptomic analysis of all identified intrahepatic macrophages revealed two subpopulations of *Clec4f*^*+*^*Timd4*^+^*Clec1b*^+^*Vsig4*^+^ KCs^13,14^ (KC1 and KC2) as previously described^15,16^, along with two subpopulations of *Cx3cr1*^+^ monocyte-derived macrophages (MoMac1 and MoMac2) and one population of *Itgax*^+^*Cd209a*^+^*Clec9a*^+^ cDCs (Fig. 2d, Extended Data Fig. 5e–g and Supplementary Table 6). Data integration of the mouse and human LM cells revealed similarities between the murine KC1 and the human LM1, sharing subcluster-specific conserved genes including *C1QA*, *C1QB*, *C1QC*, *VSIG4*, *ITM2B*, *CD81*, *SLC40A1*, *CD163* and *MRC1* (Fig. 2e, Extended Data Fig. 5h, i and Supplementary Tables 3 and 7). The murine population MoMac1 shared similarities with the human population LM3 in which genes such as *ITGAL* and *SPN* were conserved between mice and humans. The murine population MoMac2 and the human population LM4 expressed conserved genes including *LYZ* and *PLAUR*. Interestingly, although the human LM2 cells partially overlapped with the murine KC1 population and shared the expression of some general genes, no LM2-specific genes were conserved in the murine LM cell populations, suggesting that LM2 cells might be specific to humans. Some of the cells in the murine population KC2 overlapped with multiple human LMs, while a subcluster of KC2 did not overlap with any specific human population (Extended Data Fig. 5h, i). In contrast, in each human–mouse matched LM cell subset, many of the expressed genes were specific for either species (Extended Data Fig. 6a), highlighting conserved differences between human and mice. Overall, this suggests that the landscape of LM cell populations differs between mice and humans.

In addition, extensive literature has reported that the majority of macrophages in homeostatic murine livers are self-maintaining resident KCs with an embryonic origin^17–21^. We therefore analyzed the proportion of each macrophage subset in the human liver using flow cytometry, as scRNA-seq does not always reflect the true proportions of cell types within a tissue. As the liver is also highly vascularized, we analyzed the proportions in both perfused and non-perfused livers to be able to discriminate between liver resident cells and circulating monocytes. Flow cytometric analysis confirmed the presence of all four LM cell populations in both perfused (resected) and non-perfused (healthy donor) livers, with the majority being LM4 in both cohorts (29.2% in perfused and 68.5% in non-perfused livers) (Fig. 2f and Extended Data Fig. 6b). To further validate this observation, we used resected livers and compared the proportion of LM cells in the digested tissue to LM cells present in the intrahepatic blood collected from the same liver during the perfusion (Fig. 2g). The LM4 cells were detected in both the intrahepatic blood (65.9%) and the liver parenchyma (29.2%), while the LM3 cells were predominantly observed in the blood (7.3% in intrahepatic blood, 1.9% in liver), suggesting that both of these populations have a monocytic origin (Fig. 2h and Extended Data Fig. 6b). In addition, these analyses indicated that the LM4 cluster might also contain some blood monocytes, although these could also be recently recruited cells that were released from the intrahepatic vessels during the perfusion. In contrast, the LM2 cells were mainly identified in the liver tissue (18.8% in liver, 1.4% in intrahepatic blood), confirming their tissue residency (Fig. 2h). These differences in proportions of LM cell populations between mice and humans could be either environment specific or species specific^22^. To test this, we analyzed the proportion of LM subsets in laboratory wildling mice that have wild (rich and diverse) microbiota typically found in the wild that better reflects the human immune situation^23,24^. Although the total proportion of LM cells remained unchanged, wildling mice had lower proportions of KCs (57%) and higher proportions of recruited capsular macrophages (27%) compared with control mice (84% KCs and 4% capsular macrophages) housed in specific pathogen-free (SPF) conditions (Fig. 2i and Extended Data Fig. 6c–e). These data suggest that humans and mice have different LM compositions, likely due to both species and environmental differences.

### Human LM cells are mostly monocyte-derived

**Fig. 3:**
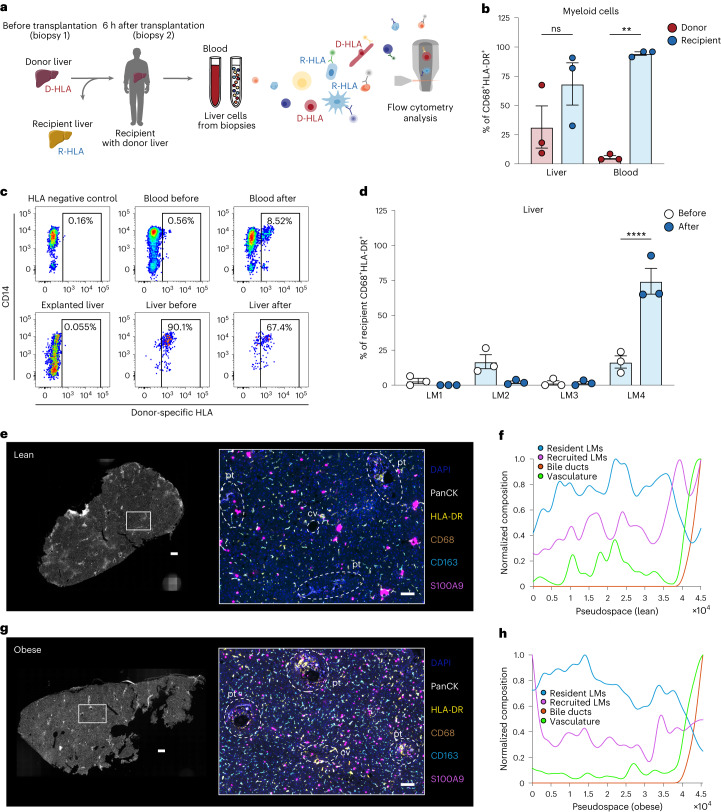
Human LM cells are mostly monocyte-derived. **a**, Experimental outline: liver biopsies and peripheral blood samples were collected before and 6 h after liver transplantation (*n* = 3 per group). Liver cells and PBMCs were then isolated and used for flow cytometric analysis to assess the proportion of recipient-derived and donor-derived cells. D-HLA, donor HLA; R-HLA, recipient HLA. **b**, Proportion of donor-derived or recipient-derived macrophages among all living CD45^+^CD68^+^HLA-DR^+^ myeloid cells after transplantation (*n* = 3 per group; blood donor versus recipient, *P* = 0.0024). **c**, Representative analysis of proportion of donor-derived cells as assessed by flow cytometric staining for donor-specific HLA. **d**, Proportion of LM subsets among all living CD45^+^CD68^+^HLA-DR^+^ myeloid cells in livers before (recipient, *n* = 3) and after (donor, *n* = 3) transplantation (LM4 before versus after, *P* < 0.0001). LM1 was defined as CD14^+^CD16^+^CD206^+^S100A9^−^, LM2 as CD14^+^CD16^−^CD206^+^S100A9^−^, LM3 as CD14^+^CD16^+^CD206−S100A9^+^ and LM4 as CD14^+^CD16^−^CD206^−^S100A9^+^. **e**, Representative images of lean human livers imaged using PhenoCycler, displaying the imaged tissue (left) and region of interest highlighting six markers (right; DAPI, PanCK, HLA-DR, CD68, CD163 and S100A9) that are colored according to the panel on the right. Regions containing portal tracts (pt) and central vein (cv) are highlighted in the image by dashed white lines. Images are representative of two individuals. Scale bar, 400 µm (left; entire tissue) and 100 µm (right; region of interest). **f**, Pseudospace plot visualizing the composition of resident and recruited myeloid cells sorted by tissue regions containing bile ducts (sorted to the right) in one lean donor. **g**, Representative images of livers of humans with obesity imaged using PhenoCycler. Images are representative of two individuals. Scale bar, 400 µm (left; entire tissue) and 100 µm (right; region of interest). **h**, Corresponding pseudospace plot. Data are presented as mean ± s.e.m. *P* values were calculated by two-way ANOVA with adjustment for multiple comparisons. **P < 0.01; ****P < 0.0001. Illustrations in **a** were partly created using components adapted from Servier Medical Art, provided by Servier, licensed under a Creative Commons Attribution 3.0 unported license. Source data

**Extended Data Fig. 7:**
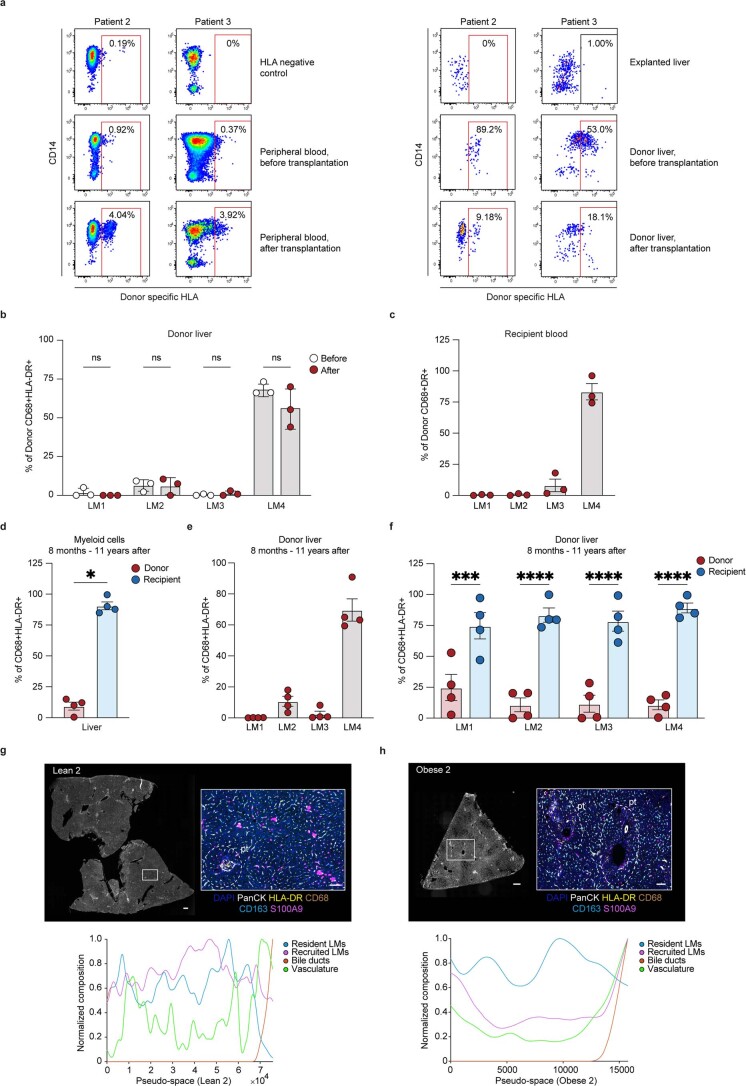
Characterisation of human liver myeloid cell turnover and tissue localisation. a, Proportion of donor-derived cells in the two other patients after undergoing transplantation, as assessed by flow cytometric staining for donor-specific HLA. b, Proportion of donor-derived LM subsets in the liver (donor, n=3) before and after (donor, n=3) transplantation into the recipient. c, Proportion of donor-derived LM subsets in the blood (recipient, n=3) after transplantation. d, Proportion of donor- or recipient-derived myeloid cell subsets among all live CD45^+^CD68^+^HLA-DR^+^ myeloid cells 8 months or 11 years post-transplantation (n=4; P=0.0286). e, Proportion of human LM subsets among all live CD45^+^CD68^+^HLA-DR^+^ myeloid cells in individuals 8 months or 11 years post-transplantation (n=4). LM1 was defined as CD14^+^CD16^+^CD206^+^, LM2 as CD14^+^CD16^-^CD206^+^, LM3 as CD14^+^CD16^+^CD206^-^, and LM4 as CD14^+^CD16^-^CD206^-^. f, Proportion of donor- or recipient-derived cells for each LM subset in individuals 8 months or 11 years post-transplantation (n=4; LM1 donor vs recipient P=0.0003 and LM2-LM4 donor vs recipient P<0.0001). g-h, Top: Images of lean human liver (Lean 2) or obese human liver (Obese 2) imaged with Phenocycler, displaying the imaged tissue (left) and region of interest highlighting six markers that are coloured according to the panel below. Regions containing portal tracts (pt) and central vein (cv) are highlighted in the image by dashed white lines. Scale bar, 400µm (left; entire tissue) and 100µm (right; region of interest). Bottom: Pseudo-space plot visualising the composition of resident and recruited macrophages sorted by tissue regions containing bile ducts (sorted to the right). Data are presented as mean ± s.e.m. P values were calculated by two-way ANOVA with adjustment for multiple comparisons (b, f) or by two-tailed Mann-Whitney test (d). *p < 0.05, ***p < 0.001, and ****p < 0.0001. Source data

In comparison with mice, our data suggested that the largest population of human liver myeloid cells, LM4 cells, was characterized by a transcriptional signature of monocyte-derived infiltrating macrophages. Studying macrophage ontogeny in humans is a challenging endeavor due to the lack of fate-mapping tools. To investigate the origin and turnover of human LM cells, we therefore analyzed the replenishment of liver myeloid cells by circulating monocytes in individuals undergoing liver transplantation (Table 2). Liver biopsies were collected before transplantation of the donor liver into the recipient, and at 6 h after transplantation. Blood samples and liver biopsies from the explanted liver were also collected, and liver cells could be distinguished as being either donor-derived or recipient-derived by staining the cells for the donor-specific human leukocyte antigen (HLA) type (Fig. 3a). Analyzing the composition of donor versus recipient myeloid cells in the donor liver revealed a significant infiltration of myeloid cells, with 68% of all myeloid cells in the liver having a recipient origin at 6 h after transplantation (Fig. 3b, c and Extended Data Fig. 7a). Next, we investigated the turnover of each LM subpopulation after transplantation and determined that the majority of recipient-derived myeloid cells that had infiltrated and populated the liver were LM4 cells (Fig. 3d). Donor LM4 cells appeared to decrease in proportion after transplantation (not significant) and could be detected in the circulation of the recipient (Extended Data Fig. 7b, c).

**Table 2:** Clinical characteristics of patients included in the study: cohort 2

Clinical parameter	Recipient (*n* = 3)	Donor (*n* = 3)
Age, years (mean, range)	37 (27–47)	61 (58–66)
Sex, female/male (*n*)	2/1	2/1
BMI, kg m^−^^2^, (mean, range)	21 (19–24)	25 (22–31)

Because the transplantation itself could affect the results of LM cell replenishment at a time point as early as 6 h after transplantation, we compared these results to data from a previously published study in which macrophages were investigated years after transplantation^25^. As this study did not analyze the pattern of turnover within different liver macrophage subsets, we analyzed the previously published flow cytometric data to examine the four LM cell subsets. This revealed that at 8 months (*n* = 2) or 11 years (*n* = 2) after transplantation, the majority of myeloid cells in the liver (90.6%) were derived from infiltrating monocytes (recipient-derived) (Extended Data Fig. 7d–f). Despite a small residual proportion of cells of donor origin still present in each population, these results highlight the general monocytic origin of all human LM cells. Taken together, these two sets of results (short-term and long-term after transplantation) suggest that most LM cells are monocyte-derived but have different turnover kinetics, with LM4 being the most rapidly replaced following transplantation (Fig. 3d and Extended Data Fig. 7f). These findings might be the result of the ischemia–reperfusion injury associated with transplantation, leading to a heightened frequency of infiltration of monocyte-derived cells to the liver and an increased liver myeloid cell turnover compared with what would have been observed in the steady state, especially at the early time point. However, the data derived from the long-term liver samples after transplantation in combination with our scRNA-seq analyses still suggest that the majority of LM cells in adult human individuals are monocyte-derived macrophages.

**Extended Data Fig. 8:**
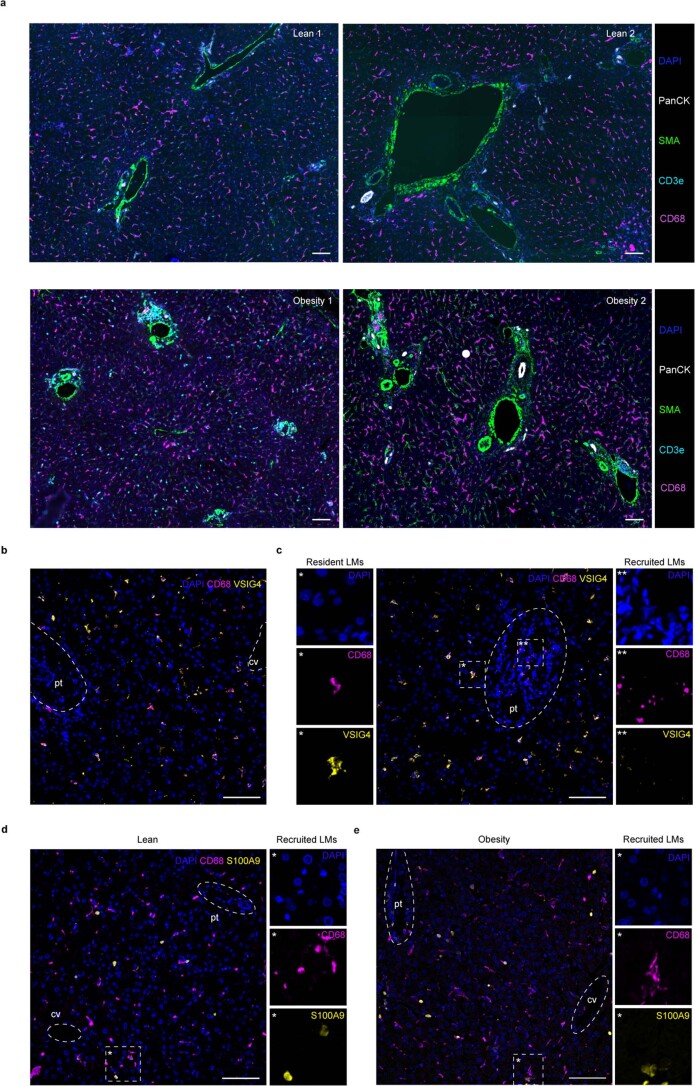
Characterisation of human liver myeloid cell localisation. a, Representative immunofluorescence images of human livers from lean (n=2) and individuals with obesity (n=2) imaged with Phenocycler, displaying region of interest highlighting five markers (DAPI, PanCK, SMA, CD3e, CD68) that are coloured according to the panel to the right. Scale bar, 100µm. b-c, Representative immunofluorescence image of tissue localization of resident (CD68^+^VSIG4^+^) and recruited (CD68^+^VSIG4^-^) myeloid cells in human livers. Regions containing portal tracts (pt) and central vein (cv) are highlighted in the image by dashed white lines. Images are representative of 4 individuals. Scale bar, 100µm. d-e, Representative immunofluorescence image of tissue localization of recruited (CD68^+^S100A9^+^) myeloid cells in livers from lean and individuals with obesity. Regions containing portal tracts (pt) and central vein (cv) are highlighted in the image by dashed white circles. Scale bar, 100µm. Images are representative of 5 lean individuals and 4 individuals with obesity. LMs, liver myeloid cells; cv, central vein; pt, portal tract.

Next, we evaluated the tissue distribution of macrophages in the liver using spatial proteomics, during which sections of liver tissues were stained with a panel of directly conjugated antibodies against immune populations and other liver cells. Interestingly, this analysis identified a small proportion of recruited myeloid cells (CD68^+^S100A9^+^) that localized in periportal areas in lean individuals (Fig. 3e and Extended Data Fig. 7g). Pseudospace analysis was further performed to visualize the spatial organization of liver cells along a defined linear axis. Pseudospace analysis in lean individuals confirmed the increased composition of vascular cells near the portal tract^4^ and revealed that the ratio of recruited LM cells was either evenly distributed or higher closer to portal tracts (defined by the identification of PanCK^+^ biliary cells and SMA^+^ portal tract vessels). In contrast, resident LM cells (CD68^+^CD163^+^) decreased in number near the portal tract (Fig. 3f and Extended Data Figs. 7g and 8a). In individuals with obesity, a slightly increased number of recruited LM cells were observed in areas furthest away from the portal tracts (Fig. 3g,h and Extended Data Figs. 7h and 8a), likely corresponding to pericentral areas (surrounding the central vein). These particular localizations have previously been associated with infiltrating monocytes differentiating into macrophages (denoted as transitioning monocytes) in both mice and humans^4,26^. Nonetheless, the majority of liver myeloid cells (HLA-DR^+^CD68^+^) in both lean individuals and individuals with obesity were localized to the zones between the portal tracts and central veins, irrespective of their phenotype (resident or recruited) (Extended Data Fig. 8a). These observations were further validated by immunofluorescence microscopy in a second cohort of lean patients and patients with obesity (Table 3 and Extended Data Fig. 8b–e).

**Table 3:** Clinical characteristics of patients included in the study: cohort 3

Clinical parameter	Lean (*n* = 6)	Obesity (*n* = 5)
Age, years (mean, range)	63 (27–77)	67 (58–75)
Sex, female/male (*n*)	2/4	2/3
BMI, kg m^−^^2^, (mean, range)	23 (21–25)	34 (32–38)*

*Average is from four individuals.

### Turnover of resident LM cells in obesity

**Extended Data Fig. 9:**
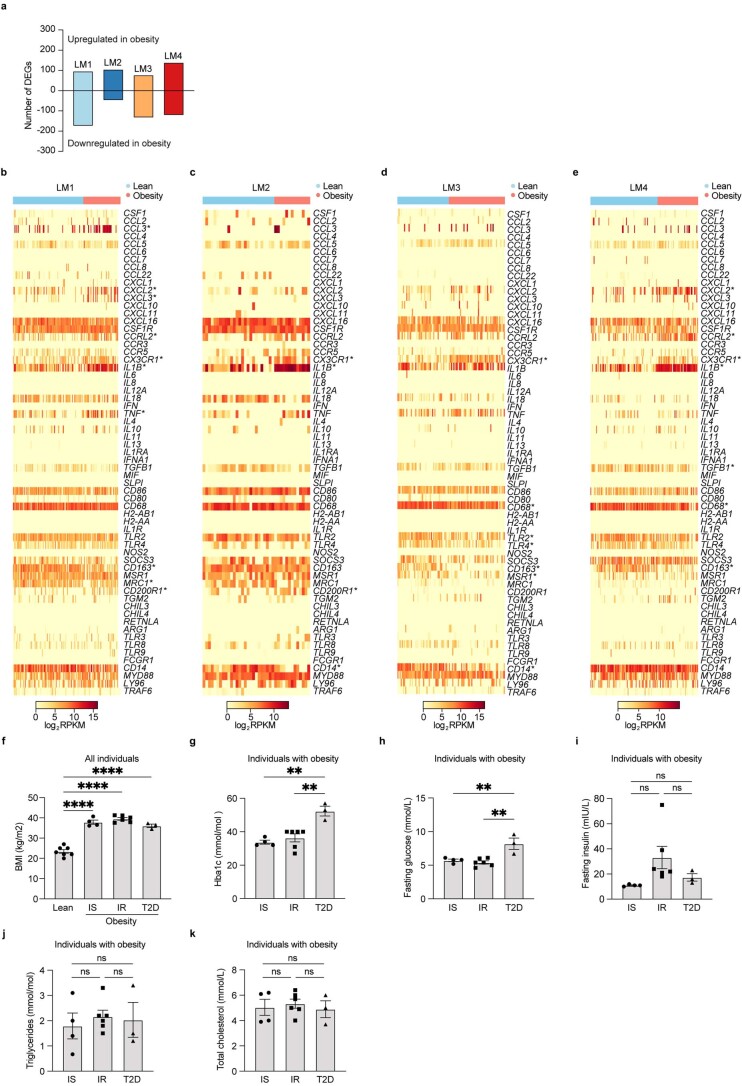
Characterisation of liver myeloid cells in obesity. a, Number of significantly regulated genes with obesity in each LM cell subpopulation (Supplementary Table 9). b-e, Heatmap of gene expression (log_2_ RPKM) of chemokines, chemokine-receptors, cytokines, M1 and M2 markers, and pattern recognition receptors in LM cell populations with obesity. Symbol indicates significantly regulated genes (adjusted P < 0.01). f, Body mass index (BMI) of lean individuals (n=7) and individuals with obesity (n=13) (lean vs IS P<0.0001, lean vs IR P<0.0001 and lean vs T2D P<0.0001). g, Haemoglobin A1c (HbA1c) levels in patients with obesity (n=13; IS vs T2D P= 0.0014 and IR vs T2D P=0.0024). h, Fasting glucose (IS vs T2D P=0.0057 and IR vs T2D P=0.0015) and i, insulin levels in patients with obesity (n=13). j, Triglyceride levels in the circulation of patients with obesity (n=13). k, Total cholesterol levels in patients with obesity (n=13). Data are presented as mean ± s.e.m. P values were calculated by one-way ANOVA with adjustment for multiple comparisons (f-k). **p < 0.01 and ****p < 0.0001. DEGs, differentially expressed genes; Reads per kilobase of exon model per million mapped reads, Reads Per Kilobases Million; IS, insulin sensitivity; IR, insulin resistance; T2D, type 2 diabetes; BMI, body mass index; ns, not significant. Source data

To understand the role of human LM populations during the development of obesity and metabolic disease, we compared the transcriptomic profiles of lean individuals and individuals with obesity. We observed that all four subpopulations were highly affected by obesity-associated NAFLD, despite the early stage of the disease (Extended Data Fig. 9a and Supplementary Table 8). Gene ontology analysis of significantly upregulated genes for each individual subpopulation in states of obesity revealed an expected upregulation of genes involved in ‘immune responses’ in all subpopulations (Supplementary Tables 8–10). However, the regulation of inflammatory genes seemed to be restricted to a small number of genes (for example, *CX3CR1* and *IL1B*), as most genes associated with inflammation remained unchanged, indicating an overall subtle inflammatory response during obesity in humans (Extended Data Fig. 9b–e and Supplementary Table 8). Moreover, although the recently recruited monocyte-derived LM4 cells were mildly pro-inflammatory compared with the other LM cells (Extended Data Fig. 3h, i and Supplementary Tables 2, 3 and 8), this phenotype was not exacerbated during obesity-associated NAFLD. These data confirm at the single-cell resolution that the LM cell inflammatory status is not dramatically affected by obesity^27^.

**Fig. 4:**
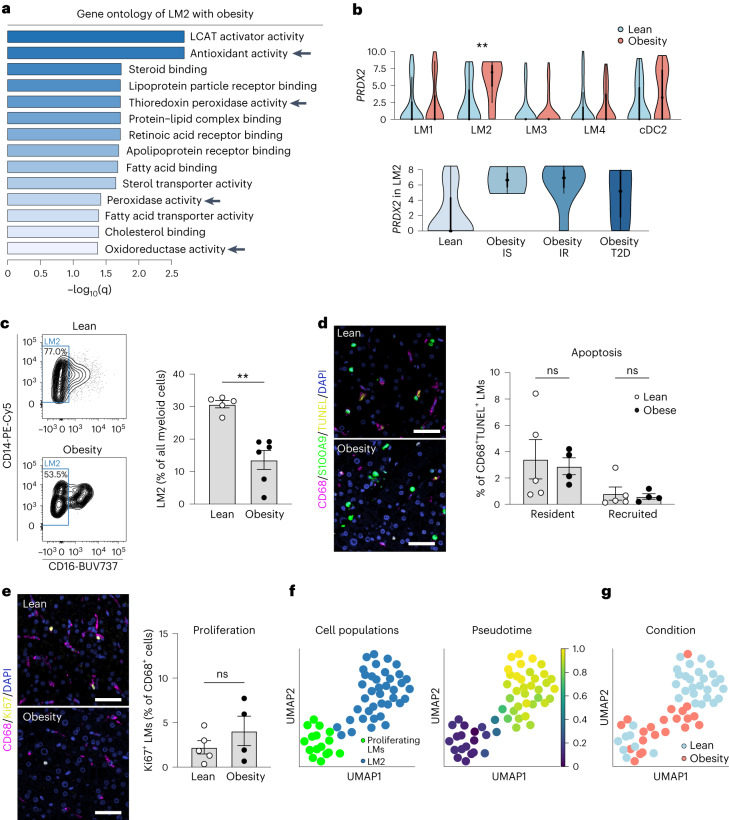
A distinct population of LM cells expresses increased levels of antioxidative genes. **a**, Gene ontology analysis of enriched molecular functions in LM2 with obesity compared with lean. LCAT, lecithin–cholesterol acyltransferase. **b**, Gene expression distribution (log_2_(RPKM)) of *PRDX2* in lean and obese LM subpopulations (top) or in LM2 stratified by obesity states (bottom). Dot indicates the median expression, thick line indicates the interquartile range, and thin line displays 1.5× interquartile range (LM2 lean versus obesity, *P* = 0.0025). IS, insulin sensitivity; IR, insulin resistance. **c**, Representative analysis of proportion of human LM2 cells among all myeloid cells (live CD45^+^CD14^+^HLA-DR^+^ cells; left) and proportion of LM2 in lean individuals (*n* = 5) and individuals with obesity (*n* = 6; right) (lean versus obese, *P* = 0.0043). **d**, Representative immunofluorescence images of CD68 (purple), S100A9 (green) and TUNEL (yellow) in human livers from lean individuals (*n* = 5) and individuals with obesity (*n* = 4) (left), and quantification of apoptotic CD68^+^S100A9^−^ resident and CD68^+^S100A9^+^ recruited LM cells (right). Scale bar, 50 µm. **e**, Representative immunofluorescence images of CD68 (purple) and Ki67 (yellow) in human livers from lean individuals (*n* = 5) and individuals with obesity (*n* = 4) (left), and quantification of proliferating CD68^+^ LM cells (right). Scale bar, 50 µm. **f**, UMAP visualization of proliferating macrophages and LM2 cells colored by cell cluster (left) and by differentiation of proliferating myeloid cells to the LM2 cluster from pseudotime analysis (right). **g**, UMAP visualization of proliferating macrophages and LM2 cells colored by condition. Data are presented as mean ± s.e.m. *P* values were calculated by one-way (**b**) or two-way (**d**) ANOVA with adjustment for multiple comparisons or by two-tailed Mann–Whitney *U*-test (**c**, **e**). **P < 0.01. Source data

In contrast, the resident LM2 cells exhibited upregulated expression of genes involved in ‘detoxification’ and ‘antioxidant response’ pathways, including the antioxidant gene *PRDX2* (Fig. 4a, b and Supplementary Tables 8–10). We then further categorized the patients with obesity into three groups: insulin sensitive, insulin resistant, and patients diagnosed with type 2 diabetes (T2D) (Supplementary Table 11). Interestingly, *PRDX2* was upregulated in both insulin-sensitive and insulin-resistant patients, suggesting obesity as a major driver of *PRDX2* upregulation (Fig. 4b). The expression of *PRDX2* was lower in patients with T2D with more severe metabolic impairment (Extended Data Fig. 9f–k).

**Extended Data Fig. 10:**
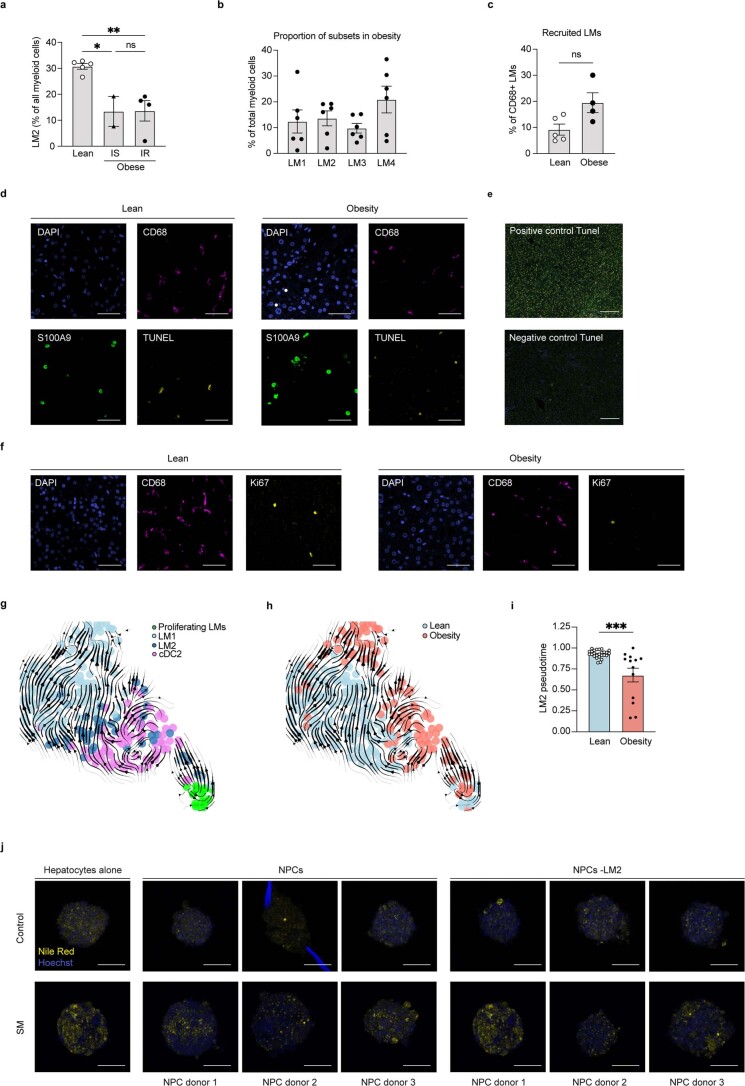
Characterisation of LM2 in obesity-induced metabolic disease. a, Proportion of LM2 cells among all myeloid cells (live CD45^+^CD14^+^HLA-DR^+^ cells) in livers from lean individuals (n=5), individuals with obesity and IS (n=2) and individuals with obesity and IR (n=4) (lean vs obesity and IS P=0.0193 and lean vs obesity and IR P=0.0063). b, Proportion of LM cell populations among all myeloid cells (live CD45^+^CD14^+^HLA-DR^+^ cells) in livers from individuals with obesity (n=6). c, Quantification of CD68^+^S100A9^+^ recruited LM cells from immunofluorescent staining’s of livers from lean individuals (n=5) and individuals with obesity (n=4). d, Individual channels of the representative immunofluorescence image included in Fig. 4d. Scale bar, 50µm. Images are representative of 5 lean individuals and 4 individuals with obesity. e, Representative immunofluorescence images of positive and negative control for the Tunel assay. Images are representative of 5 lean individuals and 4 individuals with obesity for the negative control and one lean individual and 1 individual with obesity for the positive control. Scale bar, 50µm. f, Individual channels of the representative immunofluorescence image included in Fig. 4e. Scale bar, 50µm. Images are representative of 5 lean individuals and 4 individuals with obesity. g, RNA velocity plot displaying proliferating cells and the myeloid cell cluster 1 consisting of resident LM cells and cDC2s coloured by cell population or h, by lean and obese state. Each arrow indicates the direction and progression of transcriptional states. i, Pseudotime analysis comparing differentiation of lean (n=26) and obese (n=13) LM2 cells (P=0.0002). j, Representative immunofluorescence image of liver spheroids stained for lipids using Nile red. Scale bars, 100µm. Images are representative of multiple liver spheroids from 1 hepatocyte donor and 3 NPC donors. Data are presented as mean ± s.e.m. P values were calculated by one-way ANOVA with adjustment for multiple comparisons (a, I) or by two-tailed Mann-Whitney test (c, i). *p < 0.05, **p < 0.01, and ***p < 0.001. IS, insulin sensitivity; IR, insulin resistance; LMs, liver myeloid cells; NPC, non-parenchymal cells; ns, not significant; SM, steatogenic media; ns, not significant. Source data

Considering the paradoxical regulation of *PRDX2*, a protective gene in obesity, we analyzed the proportion of LM2 and discovered it to be decreased in patients with obesity, independently of insulin resistance, compared with lean individuals (Fig. 4c and Extended Data Fig. 10a). Studies from mice have reported that resident liver macrophages decrease in number during more severe stages of liver disease such as NASH^13,28–30^, and this is suggested to be due to impaired self-renewal or loss of identity^13,30^. Interestingly, when comparing the composition of LM subsets in individuals with obesity (Extended Data Fig. 10b) with that of the healthy donor livers (Fig. 2f), changes in the proportion of LM subsets with obesity were evident. Whereas LM4 was by far the largest population in healthy donor livers (68.5%), LM4 cells comprised only 20.9% of all myeloid cells in individuals with obesity. Quantification of immunofluorescent staining of recently recruited (CD68^+^S100A9^+^) myeloid cells did not reveal any significant differences in monocyte recruitment between lean individuals and individuals with obesity (Extended Data Fig. 10c).

Because LM cell recruitment did not seem to be affected by obesity, to further investigate the mechanism underlying the decrease in LM2 cell number during obesity, we assessed apoptosis (terminal deoxynucleotidyl transferase-mediated dUTP nick end labeling (TUNEL) assay) and proliferation (Ki67 staining) in both recently recruited and resident myeloid cells. Notably, apoptosis and proliferation were similar in both fractions (Fig. 4d, e and Extended Data Fig. 10d–f), leading us to speculate that rather than a change in the number of LM2 cells, their maturation might be affected by obesity, resulting in a diminished detection using flow cytometric markers. To test this hypothesis, we analyzed the pattern of differentiation of LM2 during obesity-associated NAFLD using our scRNA-seq data. First, RNA velocity revealed a pattern of differentiation from a subset of proliferating cells (proliferating myeloid cells) to LM2 and cDC2 cells (Extended Data Fig. 10g, h). We then further analyzed the LM2 differentiation path using pseudotime and confirmed the differentiation pattern from proliferating myeloid cells to LM2 (Fig. 4f). The later stages of pseudotime contained a larger proportion of cells from lean individuals, whereas the earliest stages contained more cells from individuals with obesity (Fig. 4g and Extended Data Fig. 10i). This suggests that LM2 cells from patients with obesity might be less mature than cells from lean individuals and could explain the lower proportion of LM2 cells at these early stages of obesity-induced NAFLD. Although additional work (requiring the development of novel tools for human fate-mapping) is needed to clearly define the pattern of differentiation of LM2 in the lean and obese states, these in silico data in combination with our flow cytometric analyses suggest that the decreased proportion of LM2 cells in obesity and NAFLD could be the result of an alteration of their differentiation, rather than their recruitment, death or propagation.

### Resident LM cells reduce oxidative stress

**Fig. 5:**
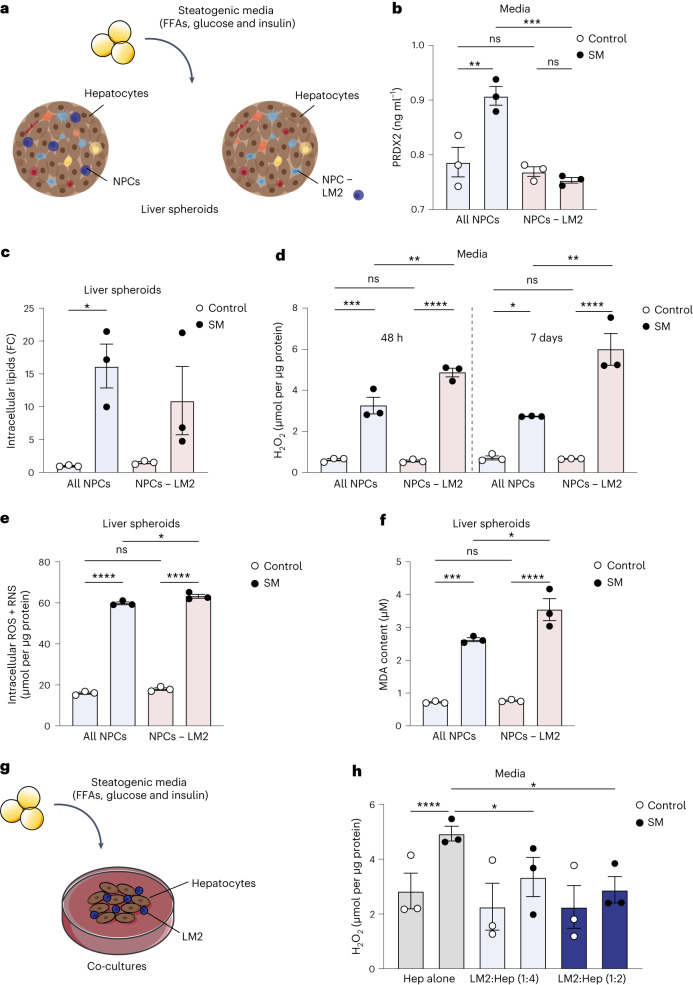
LM2 is protective by reducing oxidative stress associated with obesity. **a**, Experimental outline: human liver spheroids of hepatocytes and NPCs or with hepatocytes and NPCs where LM2 have been depleted (NPCs − LM2) using FACS were treated with high levels of FFAs, glucose and insulin (steatogenic media, SM) for 1 week. **b**, Protein quantification of PRDX2 levels in the media of liver spheroids after 7 days of treatment with SM (SM all NPCs versus NPCs − LM2, *P* = 0.0008; all NPCs SM versus control, *P* = 0.0040). **c**, Quantification of intracellular lipids in liver spheroids upon treatment with SM for 7 days (all NPCs SM versus control, *P* = 0.0339). FC, fold change. **d**, ROS (H_2_O_2_) content in media after 48 h and 7 days of treatment with SM (all NPCs SM versus control, *P* = 0.0002 (48 h) and *P* = 0.0263 (7 days); NPCs − LM2 SM versus control, *P* < 0.0001 (48 h) and *P* < 0.0001 (7 days); SM all NPC versus NPCs − LM2, *P* = 0.0050 (48 h) and *P* = 0.0016 (7 days)). **e**, Intracellular ROS and RNS in liver spheroids after 7 days of treatment with SM (all NPCs SM versus control, *P* < 0.0001; NPCs − LM2 SM versus control, *P* < 0.0001; SM all NPC versus NPCs − LM2, *P* = 0.0415). **f**, Lipid peroxidation by-product (MDA) content in liver spheroids after 7 days of treatment with SM (all NPCs SM versus control, *P* = 0.0002; NPCs − LM2 SM versus control, *P* < 0.0001; SM all NPC versus NPCs − LM2, *P* = 0.0213). **g**, Experimental outline: human primary hepatocytes were co-cultured with LM2 cells at a 1:2 or 1:4 ratio (LM2:Hep) and treated with SM for 48 h. **h**, ROS (H_2_O_2_) content in media after treatment with SM (Hep alone SM versus control, *P* < 0.0001; SM Hep alone versus LM2:Hep (1:4), *P* = 0.0346; SM Hep alone versus LM2:Hep (1:2), *P* = 0.0243). *n* = pooled liver spheroids from 1 hepatocyte donor and 3 NPC donors (**a**–**f**) or from 3 hepatocyte donors and 1 NPC donor (**g**, **h**). Data are presented as mean ± s.e.m. *P* values were calculated by one-way (**b**–**f**) or two-way (**h**) ANOVA with adjustment for multiple comparisons. *P < 0.05; **P < 0.01; ***P < 0.001; ****P < 0.0001. Illustrations in **g** were partly created using components adapted from Servier Medical Art, provided by Servier, licensed under a Creative Commons Attribution 3.0 unported license. Source data

Considering the unexpected upregulation of antioxidant gene expression in LM2 during obesity, we further investigated their role during the progression of metabolic liver disease. First, we used an in vitro model of human hepatic 3D spheroid cultures in which primary human liver cells retain their proteomic and metabolomic phenotypes for multiple weeks^31,32^. Primary human hepatocytes and NPCs from three different donors were cultured as spheroid microtissues together with pathophysiological levels of free fatty acids (FFAs), glucose and insulin in order to emulate liver steatosis, oxidative stress and insulin resistance associated with obesity and NAFLD (Supplementary Table 12; donor 1)^33,34^. Hepatic spheroid cultures were comprised of either hepatocytes and all NPCs, or of hepatocytes and NPCs from which LM2 cells had been selectively depleted using antibodies (Fig. 5a and Extended Data Fig. 4i).

PRDX2 can be secreted in response to blunt oxidative stress^35,36^. However, the role of LM-derived PRDX2 during oxidative stress associated with NAFLD in humans has not been previously described. Levels of PRDX2 were significantly increased in the media of liver spheroids with all NPCs cultured in steatogenic media, whereas they unexpectedly remained unchanged in liver spheroids lacking LM2 cells. This suggests that most secreted PRDX2 in obesity originates from LM2 cells (Fig. 5b). Although LM2 depletion had no significant effect on lipid accumulation (Fig. 5c and Extended Data Fig. 10j), it significantly increased extracellular reactive oxygen species (ROS) in spheroids treated with steatogenic media (Fig. 5d). Furthermore, the levels of intracellular ROS and reactive nitrogen species (RNS) were only slightly increased, suggesting a selective capacity of LM2 to buffer extracellular ROS (Fig. 5d, e). Enhanced lipid peroxidation as a result of oxidative stress has been previously associated with obesity and can lead to both oxidative damage and cell death^37–39^. Consistently, depletion of LM2 also significantly increased lipid peroxidation as measured by the concentration of the lipid peroxidation by-product malondialdehyde (MDA) (Fig. 5f). Since oxidative stress can be decoupled from steatosis^33^, our results suggest that LM2 reduced ROS levels in the media of liver spheroids via the release of PRDX2, independently of lipid accumulation. To further validate these observations, we performed co-cultures of primary human hepatocytes from three different donors together with sorted LM2 cells at two different concentrations (Fig. 5g, Extended Data Fig. 4i and Supplementary Table 12; donors 2–4). The addition of LM2 cells significantly decreased extracellular ROS in co-cultures treated with steatogenic media (Fig. 5h). This further supports the importance of the resident LM2 cells in maintaining reduction–oxidation (redox) balance in the liver microenvironment to protect against obesity-associated oxidative stress.

## Discussion

Beyond characterizing LM cell heterogeneity in individuals with obesity, our study unveiled striking differences in the origin and replenishment of human liver myeloid cells, which has considerable implications for the interpretation of immunological data from murine NASH models. Whereas only a minor fraction of liver macrophages is monocyte-derived during steady state in mice, the majority of human liver myeloid cells are monocyte-derived cells in both lean individuals and individuals with obesity. Studying the turnover of liver myeloid cells in individuals undergoing liver transplantation using markers of donor–recipient mismatch revealed that different LM cell populations have distinct turnover kinetics. Some LM cell populations are rapidly replaced following transplantation (hours after transplantation), while others are more slowly replaced over time (months to years after transplantation). Human livers also contained a distinct population of resident myeloid cells co-expressing markers of both macrophages and dendritic cells. These could be the recently described population of DC3s because they express both macrophage and DC markers^40,41^; however, in-depth analysis would be required to understand the identity and origin of this population. These cells also expressed high levels of genes regulating oxidative and metabolic stress in obesity, in particular the antioxidant *PRDX2*. Moreover, their proportion decreased with obesity due to the inability to fully differentiate into mature macrophages. Our functional analyses demonstrated that the PRDX2 protein was upregulated and released into the media in our human in vitro 3D liver cultures in response to treatment with steatogenic media. Importantly, experimental removal of the same LM cell population was sufficient to exacerbate both the release of oxidative stress species and of lipid peroxidation in 3D microtissues. In contrast, addition of the same population to 2D cultures with primary hepatocytes was protective against the oxidative stress induced by the steatogenic media. Because resident LM cells are protective against metabolic impairments, we envisage a cellular network whereby resident LM cells might not fully acquire their protective phenotype during obesity. In summary, our study highlights a novel cellular mechanism in which resident LM cells attenuate the burden of oxidative stress during early stages of obesity-associated metabolic disease.

## Methods

### Patients

Human adult liver samples were collected from 13 individuals with obesity (BMI, 35–42 kg m^−2^), of which five were used for scRNA-seq. The patients underwent laparoscopic Roux-en-Y gastric bypass surgery and did not have any previous history of cardiovascular disease, gastrointestinal disease, systemic illness, alcohol abuse, coagulopathy, chronic inflammatory disease, or any clinical sign of liver damage or surgical intervention within 6 months prior to the study. Patients did not receive any low-calorie diet before the surgery, as this would influence the degree of liver steatosis and inflammation. All individuals with obesity had an HSI > 36, indicative of NAFLD, as calculated by ref. ^42^. Insulin sensitivity was assessed using the homeostatic model assessment for insulin resistance (HOMA-IR), for which four individuals with HOMA-IR < 3.5 were classified as having obesity with insulin sensitivity, and nine individuals with HOMA-IR > 3.5 were classified as having obesity with insulin resistance. Three of the patients with obesity with insulin resistance were previously diagnosed with T2D. Controls included 12 lean individuals (maximum BMI, 25) with primary or metastatic liver tumors, or from donor livers rejected for transplantation. Liver samples were collected during liver resection surgery, and only non-affected tissues were used. The lean patients used for scRNA-seq (*n* = 3) had not received chemotherapy or radiotherapy prior to surgery and had no history of T2D. For liver samples used for immunofluorescence, a second cohort of samples was collected from five individuals with obesity (BMI, 30–38 kg m^−2^) and six lean individuals (maximum BMI, 25) undergoing liver resection surgery.

For liver biopsies collected from individuals undergoing liver transplantation with HLA-mismatched liver allografts, liver samples were collected from three donor and three explanted livers. The patients underwent transplantation surgery due to primary sclerosing cholangitis, primary biliary cirrhosis or progressive familial intrahepatic cholestasis. Biopsies were collected from the donor liver before transplantation into the recipient, and at the end of the transplantation surgery. Liver biopsies were also collected from the explanted liver (from the recipient) during the transplantation surgery, after its removal. Peripheral blood samples (from the recipient) were collected at the same time as the second liver biopsy from the donor liver. Monocytes used for Cytospin were isolated from buffy coats from blood donations of healthy volunteers from the local hospital blood bank. In addition, the human hepatocytes used for 2D and 3D culture in Fig. 5 were procured from BioreclamationIVT. All of the studies involving human subjects have been planned in compliance with national legislation and the World Medical Association’s Code of Ethical Principles for Medical Research Involving Human Subjects (Declaration of Helsinki), and have been granted ethical approvals by the Regional Ethical Review Board in Sweden (Regional Ethical Committee in Stockholm, Sweden (2017/214-31, 2017/269-31, 2008/1010-31, 2006/229-31)). All individuals provided oral and written informed consent. Additionally, no compensation or any other form of payment was given to participants to ensure that samples were donated out of free will and for no other reason.

### Isolation of NPCs from human biopsies and PBMCs

Human liver samples from individuals with obesity (collected during gastric bypass surgery) and liver samples collected from transplantation surgeries were processed by mechanical dissociation before digestion with 0.25 mg ml^−1^ collagenase II (Sigma, C6885) and 0.2 mg ml^−1^ DNase I (Roche, 1010415900) at 37 °C for 30 min. Cell suspensions were filtered through a 70-µm cell strainer and centrifuged at 50 × *g* for 3 min to pellet the hepatocytes. The supernatant containing the NPCs was washed once in PBS and cryopreserved in FBS with 10% DMSO (Sigma, D2650) and stored in liquid nitrogen for subsequent analyses. Human liver samples from individuals undergoing liver resection surgery, including the samples collected from intrahepatic blood and liver tissues from the same donor, were processed by a three-step perfusion technique^43^. Liver excess sinusoidal blood (intrahepatic blood) was collected in a series of flushing steps. Livers were then subjected to several perfusion steps including enzymatic digestion with collagenase XI (Sigma). Cells were cryopreserved in FBS with 10% DMSO and stored in liquid nitrogen for subsequent analyses. Peripheral blood mononuclear cells (PBMCs) from blood samples were isolated with density gradient centrifugation. Samples were first diluted with PBS and loaded onto a Ficoll-Hypaque media solution (GE Healthcare, 17-1440-02) and centrifuged at 2,000 r.p.m. for 20 min at room temperature. The interphase ring with enriched mononuclear cells was collected and washed twice before cryopreservation for subsequent analyses.

### Mice

Mice were group-housed under SPF conditions and maintained on a 12 h/12 h light/dark cycle at 20 ± 1 °C with 50–53% humidity with ad libitum access to food and water. Male mice were used in all experiments due to the predominant use of male mice in previous studies of diet-induced obesity and metabolic disease. Four-week-old wild-type C57BL/6J mice were obtained from Charles River Laboratories and fed a high-fat diet (calories consisted of 60% fat, 20% carbohydrates and 20% proteins) (Research Diets Inc., D12492) for a total of 9 weeks, starting at 5 weeks of age. Control mice were fed a normal chow diet (Teklad Global, 2918).

S.P. Rosshart kindly provided C57BL/6NTac wildling mice and C57BL/6NTac pathogen-free control mice. C57BL/6NTac wildling mice were created through inverse germ-free rederivation as previously described^23^. Wildling mice were housed in seminatural housing conditions created by cage supplementation with natural materials including hay, compost and fomites from actual wild mice. Additionally, litters from different breeding pairs were fraternized in large cages to increase their microbial exposures in adolescence. C57BL/6NTac murine pathogen-free mice were used as control and adhered to our characterization of SPF. Male wildling and C57BL/6NTac SPF mice were 8–12 weeks old at the start of the experiment and age-matched within the experiments. All procedures were performed in accordance with guidelines approved by the Regional Ethical Committee in Stockholm (Stockholms djurförsöksetiska nämnd, Stockholms södra djurförsöksetiska nämnd and Linköpings djurförsöksetiska nämnd).

### Lipid quantifications in murine livers

Liver samples were collected and instantly frozen in liquid nitrogen. Total triglyceride content was then determined using the Triglyceride Colorimetric Assay Kit (Cayman Chemical, 10010303) following the manufacturer’s instructions. Measured triglyceride concentrations were normalized against the protein concentration of each sample, as measured by the Pierce BCA Protein Assay Kit (Thermo Fisher Scientific, 23227).

### Mouse metabolic analysis

A glucose tolerance test was performed on the day before NPC isolation. Mice were fasted for 6 h and subsequently administered with 1 mg g^−1^ glucose by intraperitoneal injection. Blood glucose levels were measured from the tail vein at defined time points using a glucometer (Accu-Chek Aviva Blood Glucose Meter, Roche Diabetes Care).

### Isolation of murine NPCs

Murine NPCs used for scRNA-seq were isolated by in vivo digestion. Anesthetized mice were first perfused with HBSS-EGTA buffer (0.5 mM EGTA in HBSS; Gibco, 14185045), followed by perfusion with collagenase II. Once digested, livers were collected and cells were released by mechanical dissociation. Cell suspensions were filtered through a 100-µm cell strainer and centrifuged at 50 × *g* for 3 min to pellet the hepatocytes. The resulting supernatant containing the NPCs was washed twice with PBS to be used for single-cell sorting. For isolation of murine NPCs from wildling mice and SPF control mice, animals were euthanized followed by perfusion with PBS. Livers were collected in PBS and then processed by mechanical dissociation before digestion with 0.25 mg ml^−1^ collagenase II and 0.2 mg ml^−1^ DNase I at 37 °C for 30 min. Cell suspensions were filtered through a 70-µm cell strainer and centrifuged at 50 × *g* for 3 min to pellet the hepatocytes. The supernatant containing the NPCs was subsequently washed twice in PBS and used for flow cytometry.

### Single-cell sorting of NPCs

Cryopreserved human NPCs were thawed, whereas murine NPCs were used freshly isolated. Cells were stained at 4 °C for 20 min with antibodies (Supplementary Table 13) and with a viability dye (Thermo Fisher Scientific) to discriminate live and dead cells. Cells were then washed and resuspended in FACS buffer (1% BSA in PBS). Sorting of samples from individuals with obesity was performed by gating on live cells (single cells). Sorting of lean samples was performed by negatively gating out dead cells, T cells, B cells and NK cells in order to unbiasedly enrich for myeloid cells. Cells were single-cell index sorted into 384-well PCR plates (FrameStar) containing 2.3 µl of lysis buffer per well (0.2% Triton X-100 (Sigma, T9284), 2 U RNase Inhibitor (Clontech, 2313B), 1 µM Oligo-dT_30_VN primer (Integrated DNA Technologies; 5′-AAGCAGTGGTATCAACGCAGAGTACT_30_VN-3′), 2 mM dNTP (Thermo Fisher Scientific, R1122) and 0.025 µl of the spike-in RNA ERCC (1:40,000) (Thermo Fisher Scientific, 4456740)), on a BD FACSAria Fusion (equipped with four lasers). Plates were stored at −80 °C until processed.

### Single-cell RNA library preparation and sequencing

Single-cell RNA libraries were prepared using the Smart-seq2 protocol^44^ with minor modifications. mRNA from single cells was converted to cDNA by adding 2.7 µl of reverse transcription mix (1x SuperScript II buffer (Invitrogen, 18064014), 1 M betaine (Sigma, B0300), 5 mM DTT (Invitrogen, 18064014), 7 mM MgCl_2_ (Thermo Fisher Scientific, AM9530G), 50 U SuperScript II reverse transcriptase (Invitrogen, 18064014), 5 U RNase Inhibitor (Clontech, 2313B), 2 µM TSO primer (Qiagen; 5′-AAGCAGTGGTATCAACGCAGAGTACATrGrG+G-3′)) and incubated in a thermal cycler (42 °C for 90 min; 10 cycles of 50 °C for 2 min and 42 °C for 2 min; then 70 °C for 15 min). cDNA was then amplified by adding 7.5 µl of PCR pre-amplification mix (1x KAPA HiFi HotStart ReadyMix (Roche, KK2602) and 0.08 µM ISPCR primer (Integrated DNA Technologies; 5′-AAGCAGTGGTATCAACGCAGAGT-3′)) and run on a thermal cycler (98 °C for 3 min; 24 cycles of 98 °C for 20 s, 67 °C for 15 s and 72 °C for 6 min; then 72 °C for 5 min). Amplified cDNA was purified using Agencourt AMPure XP beads (Beckman Coulter, A63881) at a ratio of 1:0.8 (cDNA:beads). Then, 0.5 ng of cDNA from each well were fragmented by incubation with a tagmentation mix (1x TAPS-MgCl_2_ (10 mM TAPS-NaOH, 5 mM MgCl_2_), 10% PEG 8000 (Sigma, P1458) and 150 nM Tn5 enzyme) at 55 °C for 5–7 min, followed by incubation with 0.02% SDS at room temperature for 5 min to terminate the reaction. Fragmented cDNA was barcoded with a unique combination of the Nextera v2 index primers (1:5 dilution) for each cell (Illumina, FC-131-2001 to FC-131-2004) and run together with 6 µl of amplification PCR mix (1x KAPA HiFi buffer, 0.3 mM dNTP and 0.4 U KAPA enzyme (Roche, KK2102)) on a thermal cycler (72 °C for 3 min; 95 °C for 30 s; 10 cycles of 95 °C for 10 s, 55 °C for 30 s and 72 °C for 30 s; then 72 °C for 5 min). Two microliters of libraries from each well were pooled and purified using Agencourt AMPure XP beads at a ratio of 1:0.6 (cDNA:beads). Libraries were diluted to 3 nM and sequenced on a HiSeq 3000 Sequencing System (Illumina) at 50 bp single read.

### Bioinformatic analysis of scRNA-seq data

#### Preprocessing

Raw sequencing data were demultiplexed and converted into fastq files using bcl2fastq (Illumina) with default settings. The human reads were aligned to the human genome hg38 and mouse reads to the mouse genome mm10 using STAR (v2.4.2) with default settings, and filtered for uniquely mapped reads^45^. Gene expression values were calculated as reads per kilobase transcript and million mappable reads (RPKMs) using rpkmforgenes^46^.

#### Quality control

Low-quality cells or empty wells were filtered out using the following inclusion criteria: (1) ≥50,000 sequenced reads, (2) ≥20% of reads that uniquely mapped to the genome, (3) ≥30% (human) or ≥40% (mouse) exon mapping reads annotated using RefSeq, (4) ≥500 (human) or ≥1,000 (mouse) genes with RPKM ≥ 1, and (5) <25% of mitochondrial gene expression. In addition, doublets detected by Scrublet^47^ were further removed, resulting in a total of 1,351 human and 1,470 mouse single cells for downstream analysis.

#### Cell population assignment

To identify cell populations, the top 1,000 significant genes (false discovery rate (FDR) < 0.01) with high biological variability over technical noise were used for principal component analysis (PCA) dimensionality reduction^48^. The top significant principal components (*P* < 0.05), determined by 500 random permutations based on the jackstraw approach^49^, were used for spectral clustering from the scikit-learn Python library (v0.24.2). Each cell cluster was assigned to a cell type based on the expression of classical cell-type markers from the differential expression analysis of the identified cell populations, resulting in B cells (*n* = 60), T cells (*n* = 361), endothelial cells (*n* = 12), cDC1 (*n* = 15), myeloid cells 1 (*n* = 246), myeloid cells 2 (*n* = 228), proliferating cells (*n* = 27), resident NK cells (*n* = 181), circulating NK cells and NKT cells (*n* = 211) and mast cells (*n* = 10) for human cell types; and CD4^+^ T cells (*n* = 246), erythrocytes (*n* = 29), CD8^+^ T cells (*n* = 60), NK cells (*n* = 45), B cells (*n* = 250), endothelial cells 1 (*n* = 176), endothelial cells 2 (*n* = 289), KCs (*n* = 248), monocyte-derived macrophages (*n* = 53), neutrophils (*n* = 14), hepatocytes (*n* = 38) and plasmacytoid dendritic cells (pDCs; *n* = 22) for mouse cell types. To distinguish subpopulations of the human liver macrophages, a similar analysis approach as described above was applied, with the only differences being the use of the top 500 significantly variable genes (FDR < 0.01) identified in the 474 liver myeloid cells and the use of *k*-means clustering. This resulted in the identification of four distinct subpopulations: LM1 (*n* = 133), LM2 (*n* = 112), LM3 (*n* = 96) and LM4 (*n* = 133). The LM2 cluster was further divided into LM2 (*n* = 39) and cDC2 (*n* = 73) using spectral clustering based on the expression of conventional macrophage markers (*CD68*, *CD14*, *CSF1R*, *CD163* and *VSIG4*). Subcluster analysis of the 301 mouse liver macrophages resulted in four distinct subpopulations, including KC1 (*n* = 225), KC2 (*n* = 25), MoMac1 (*n* = 18), MoMac2 (*n* = 18) and cDCs (*n* = 15). Furthermore, spectral clustering of KCs (*n* = 569) and cDC2 cells (*n* = 1,666) from two published scRNA-seq datasets^4,50^ resulted in five or four subclusters based on the 500 or 1,000 most variable genes, respectively.

#### Neural network cell-type scoring

The calculation of the probabilistic score was performed as previously described^51^, and the analysis package will be available as a machine learning-based single-cell analysis toolkit, scCAMEL (https://sccamel.readthedocs.io). In brief, a neural network classifier was built to learn the defined cell types. Before the training, cell cycle-related genes were removed, and the marker genes for each cell type were ranked. Subsequently, the ranked marker genes were log-transformed, min–max normalized, and then applied for the classifier training. The learning frame and the parameters of the neural network classifier have been listed in the previous study^51^. The classifier’s learning accuracy was inspected against epoch numbers and was estimated by *k*-fold cross-validation. The learning rate and learning epochs were decided following the maximum point when the curve achieving the accuracy plateaus. Data were visualized in a radar plot. Each predicting cell’s position is a linear combination of the probabilistic scores against all trained cell types, and the position was visualized as the relative position to all polygon vertices.

#### Differential expression analysis between defined groups

To define specific genes for each population, differentially expressed genes were identified by performing the Kruskal–Wallis test with Benjamini–Hochberg multiple testing correction on the log_2_-transformed expression data. Conover–Iman post hoc tests were performed for all possible pairwise cell population comparisons. Genes that were significantly upregulated in one cell population compared with other cell populations (post hoc adjusted *P* < 0.01, minimum fold change of 2 and at least one cell population with mean log_2_(RPKM) ≥ 3) were considered as cell population-specific upregulated genes. To identify genes differentially expressed between lean and obese conditions within the same cell population, we used one-way analysis of variance (ANOVA) with Tukey’s honestly significant difference test. Differentially expressed autosomal genes were selected based on adjusted *P* < 0.01, fold change of 2 and at least one condition with mean log_2_(RPKM) ≥ 3. Differentially expressed genes were further functionally annotated by analysis of statistically overrepresented gene ontology terms (adjusted *P* < 0.05) using goenrich.

#### scRNA-seq data integration

Integration of the 1,351 adult human NPCs to 113,063 human embryonic liver cells (accession code E-MTAB-7407)^10^ was performed using the standard workflow of Seurat (v4.0.5) multiple dataset integration^52^. The top 2,000 most variable features selected using the ‘vst’ method were used in anchor finding for dataset integration. Integration of the human (*n* = 1,351) and mouse (*n* = 1,470) NPCs, or the human (*n* = 474) and mouse (*n* = 301) liver myeloid cells were performed using the standard workflow in Seurat (v4.0.5) multiple dataset integration^52^. A total of 10,646 genes with one-to-one homologs in both species were found to be expressed by both human and mouse liver macrophages and were used for the analysis. From these, the top 2,500 most variable features selected using the ‘vst’ method were used in anchor finding for dataset integration.

#### Analysis of conservation of genes between human and mouse LM cells

A list of homologous genes between human and mouse was obtained from the Ensembl database (v92) using BioMart. Genes with multiple orthologs were excluded from the analysis. For each matched human and mice LM subset, genes with a median log_2_(RPKM) ≥ 4 in both species were considered as conserved highly expressed genes. To characterize subcluster-specific conserved genes, differential expression analysis was performed among subsets that exist in both species in order to identify conserved genes that are significantly overexpressed in one subset compared with the other, in both species.

#### Trajectory analysis

To define cell trajectories between human proliferating cells and liver resident myeloid cells, we performed RNA velocity and pseudotime analysis. As the cluster of proliferating cells expressed both macrophage markers and markers of other cell types, suggesting potential subpopulations within these cells, we first performed subcluster analysis using spectral clustering based on the top 500 variable genes. This resulted in two distinct subsets of proliferating cells, of which cluster 1 (*n* = 16) comprised proliferating myeloid cells and cluster 2 (*n* = 11) comprised other cell types. For RNA velocity analysis of the cluster of proliferating myeloid cells and myeloid cells 1, the spliced and unspliced counts for each gene were generated using velocyto (v0.17)^53^. With scVelo, the spliced and unspliced counts were further normalized and log-transformed using the scVelo package (v0.2.4)^54,55^. The top 3,000 highly variable genes based on the normalized counts were then used to compute the nearest neighbors (n_neighbors = 15) of the single cells in PCA space along with the moments (n_pc = 20), based on the resulted connectivity. The velocity estimation was based on a stochastic model that considers both the balance of unspliced to spliced mRNA levels and their covariation. In addition, to analyze the cell differentiation trajectory from proliferating myeloid cells (*n* = 16) to LM2 cells (*n* = 39), pseudotime analysis was performed based on the 848 significantly variable genes. Pseudotime was calculated by the geodesic distance along the graph using the Scanpy package (v1.7.2).

### Flow cytometric analysis of liver and blood samples

Phenotyping of human tissue and blood samples was performed on a BD LSRFortessa (equipped with five lasers), and phenotyping of murine tissues was performed on a BD Symphony A3 (equipped with five lasers) using the flow cytometer driver software (FACSDiva v8.0.1, v8.0.2 and v9.1). Human cryopreserved cells were thawed, washed once with FACS buffer (PBS, 2% FCS and 4 mM EDTA) and stained for 20 min with antibodies (Supplementary Table 13) and with a viability dye (Thermo Fisher Scientific) to discriminate live and dead cells. Murine cells were stained freshly isolated. Cells were fixed with the Foxp3/Transcription Factor Staining Kit (eBioscience, 00-5523-00) and acquired on the flow cytometer. Samples used for analysis of LM subset proportion in lean compared with obese were run unfixed. For the data in Extended Data Fig. 7d–f, proportions of human myeloid cells were analyzed from the previously published flow cytometry dataset of human HLA-mismatched liver allografts generated by ref. ^25^. All analyses were performed using FlowJo (v9.9.6, v10.5.3 and v10.8.1).

### Immunofluorescence microscopy

Liver samples were embedded in Tissue-Tek OCT compound or fixed in 4% formaldehyde at 4 °C overnight prior to embedding in paraffin. Paraffin-embedded tissues were sectioned at a thickness of 5 µm, and OCT-embedded fresh liver sections were sectioned at a thickness of 10–15 µm. Fresh liver sections were fixed in 4% formaldehyde solution at 4°C for 15 min, permeabilized in 0.1% Triton X-100 in PBS for 30 min and blocked with 5% normal goat serum (Invitrogen, 50062Z) in PBS for 1 h. Paraffin-embedded liver sections were deparaffinized in xylene (2 × 5 min) and rehydrated in ethanol (2 × 5 min in 100%, 5 min in 95% and 5 min in 70%), followed by antigen retrieval in sodium citrate buffer (10 mM sodium citrate, 0.05% Tween 20, pH 6.0) using a 2100 Antigen Retriever (Apton Biologics) and blocking in 0.1% Triton X-100 in Background Buster (Innovex Biosciences, NB306) for 30 min. All sections were incubated with primary antibodies (1:50 dilution in blocking buffer) at 4 °C overnight, followed by incubation with secondary antibodies (1:500 dilution) for 1 h at room temperature (antibodies used are listed in Supplementary Table 14). Nuclei were counterstained with DAPI (Sigma, MBD0015), and slides were mounted with ProLong Diamond Antifade Mountant (Thermo Fisher Scientific, P36961) and imaged with an Axio Observer Z1 fluorescence microscope (Axiocam 506 mono, Zeiss) at ×10 magnification.

Apoptotic cells were visualized using the In Situ Cell Death Detection Kit (Roche, 12156792910) according to the manufacturer’s instructions with minor modifications. In brief, liver sections were processed as described above, and incubated with 100 µl of TUNEL reaction mix (16.5 µl of Label enzyme and 1.7 µl of Label solution in 83.3 µl of PBS) at 37 °C for 60 min. For positive control, liver sections were treated with DNase I solution (1 mg ml^−1^ in 50 mM Tris, pH 7.5, 1 mg ml^−1^ BSA) for 10 min to induce DNA damage prior to incubation with the TUNEL reaction mix. Sections were then stained with antibodies as described above and imaged with the Axio Observer Z1 fluorescence microscope at ×10 magnification. For image quantifications, five arbitrary areas were imaged per sample and cell counting was done using ImageJ (v1.52h). For visualization purposes, images were acquired with a Nikon A1R confocal microscope (Eclipse Ti, Nikon) at ×20 magnification in z-stacks of eight images per field. For each stack, a maximum intensity z-projection was generated using ImageJ and autofluorescence was subsequently filtered from the image by creating a mask of the green channel autofluorescence that was subtracted from the Cy3 and Cy5 signals using the ImageJ subtract function. Background in the DAPI channel was filtered from the image using the ImageJ subtract background tool (rolling ball radius = 50 pixels).

### Histology and histological stainings

Paraffin-embedded livers were stained with either hematoxylin and eosin (H&E) or picrosirius red. OCT-embedded fresh liver sections were stained with Oil Red O. All sections were imaged with a Panoramic 250 Slide Scanner and opened in QuPath (v0.3.1) to save regions of interests.

### Cytospins

Cryopreserved liver NPCs were stained with FACS antibodies as described above. Cells were sorted on a Sony MA900 (equipped with three lasers) using the flow cytometer driver software (Sony Cell Sorter Software v3.1.1) into 200 µl of FACS buffer. Monocytes were isolated from buffy coats donated by healthy volunteers. PBMCs were first isolated using density gradient centrifugation as described above. From the PBMCs, monocytes were isolated using the Human Pan Monocyte Isolation Kit (Miltenyi Biotec, 130-096-537). All cytospins were performed at 300 r.p.m. for 10 min on a Cytospin 4 Centrifuge (Thermo Fisher Scientific). Cells were stained with Wright-Giemsa (Abcam, ab245888) and imaged using a Leica DM4000 B microscope. Images were taken of 8–25 fields for each sample at ×100 magnification. For visualization purposes, slide background was filtered from the image using the ImageJ (v2.9.0) subtract background tool (rolling ball radius = 25).

### Spatial proteomics by PhenoCycler

Paraffin-embedded liver sections were prepared as previously described^56,57^. Sections were run on an Akoya PhenoCycler-Fusion system (Akoya Biosciences), using the PhenoCycler driver software. In brief, 11 cycles were run with a panel of 14 antibodies and DAPI (Supplementary Tables 15 and 16), and automated imaging was done of the entire tissue region at ×20 magnification (0.5 µm per pixel). Processed images generated from the PhenoCycler were opened in QuPath (v0.3.1), and regions of interests were saved to include a scale bar. For pseudospace analysis, images were segmented using the ‘cell detection’ function in QuPath (detection channel, DAPI; requested pixel size, 0.5 µm; nucleus background radius, 6 µm; nucleus median filter radius, 0 µm; nucleus sigma, 1 µm; nucleus minimum area, 8 µm^2^; nucleus maximum area, 400 µm^2^; intensity threshold, 15; cell expansion, 5 µm). Segmented data containing the cell segmentation information, spatial position for each cell and the mean expression of each marker were exported as csv files for further analysis using the histocytometric multidimensional analysis pipeline (CytoMAP v1.4.21) in MATLAB (vR2022b)^58^. Cell phenotypes (vasculature cells, biliary cells, and resident and recruited LM cells) were defined by manual gating using the protein expression of the following markers: DAPI, PanCK, HLA-DR, CD68, CD163, S100A9 and SMA. Neighborhood analysis was performed to divide tissues into local neighborhoods (or circular areas) using the Raster scan neighborhood function (neighborhood radius = 50) and used for pseudospace analysis of the distribution of cell phenotypes across tissues, where neighborhoods were sorted based on the composition of biliary cells using default parameters.

### Liver spheroids

LM2 cells were depleted from NPC samples by sorting on a Sony MA900 (equipped with three lasers) or a BD FACSAria Fusion (equipped with four lasers). NPCs were stained with FACS antibodies as described above (Supplementary Table 13). LM2 cells were excluded by gating out CD3^−^CD19^−^HLA-DR^+^CD14^+^CD16^−^CD206^+^ cells, and non-LM2 cells were sorted into William’s Medium E (PAN-Biotech, P04-29500) supplemented with 10% FBS. Liver spheroids were formed with either all NPCs or NPCs without LM2 cells.

To form liver spheroids, NPCs were seeded together with cryopreserved primary human hepatocytes (BioreclamationIVT; donor 1 from Supplementary Table 12) at a ratio of 1:3 (NPCs:hepatocytes), with 1,500 cells per spheroid, into ultra-low attachment 96-well plates, as previously described^59^. To encourage cell aggregation, plates were centrifuged at 180 × *g* for 3 min. If cells were not well aggregated, plates were centrifuged again. Co-cultures were kept in low-glucose/insulin medium (William’s E medium (PAN-Biotech) supplemented with 5.5 mM D-glucose, 0.1 nM insulin, 2 mM L-glutamine, 100 units ml^−1^ penicillin, 100 μg ml^−1^ streptomycin, 5.5 μg ml^−1^ transferrin, 6.7 ng ml^−1^ sodium selenite, 100 nM dexamethasone and 10% FBS) for 6 days to allow spheroids to become sufficiently compact; thereafter, 50% of the medium was replaced with serum-free medium.

For treatment with FFAs, spheroids were cultured for 7 days in serum-free high-glucose/insulin medium (William’s E medium (Gibco), supplemented with 11.1 mM D-glucose, 1.7 μM insulin, 2 mM L-glutamine, 100 units ml^−1^ penicillin, 100 µg ml^−1^ streptomycin, 5.5 μg ml^−1^ transferrin, 6.7 ng ml^−1^ sodium selenite and 100 nM dexamethasone) containing 240 μM oleic acid and 240 μM palmitic acid, both previously conjugated to 10% BSA at a molar ratio of 1:5 at 40 °C for 2 h. Non-FFA-treated spheroids continued culture in serum-free low-glucose/insulin medium.

For intracellular lipid staining, spheroids were fixed in 10% formalin and stained with Nile red (2 μM) and Hoechst 33342 (1 μg ml^−1^) at room temperature for 24 h. Stained spheroids were imaged using a Zeiss LSM 880 confocal microscope. For intracellular lipid quantifications, spheroids were trypsinized and subsequently mechanically dissociated in the presence of AdipoRed Assay Reagent (Lonza, PT-7009) before fluorescence signals were measured.

### Hepatocyte and LM2 co-cultures

Cryopreserved primary human hepatocytes (BioreclamationIVT) from three different donors (Supplementary Table 12, donors 2–4) were thawed according to manufacturer’s instructions. Viable hepatocytes were seeded into 48-well plates coated with collagen solution (Sigma, C3867) in low-glucose/insulin medium (as prepared for the liver spheroids) and allowed to adhere to the plate for 1–2 h before the addition of LM2 cells. LM2 cells were sorted from cryopreserved NPCs from one donor on a Sony MA900 (equipped with three lasers) as described above. Sorted viable LM2 cells were added to the hepatocytes at a ratio of 1:2 or 1:4 (40,000 or 20,000 LM2 cells and 80,000 hepatocytes). At day 1 after seeding, co-cultures were treated with high glucose/insulin and FFAs (11.1 mM D-glucose, 1.7 μM insulin, 240 μM oleic acid and 240 μM palmitic acid) or continued culture in low-glucose/insulin medium. Supernatants and cells were collected for subsequent analyses after 48 h of treatment.

### ELISA

PRDX2 protein levels were measured using the Human Peroxiredoxin 2 DuoSet ELISA (R&D Systems, DY3489-05), following the manufacturer’s instructions.

### Lipid peroxidation and ROS measurements

Lipid peroxidation was determined by measuring the content of MDA using the Lipid Peroxidation (MDA) Assay Kit (Colorimetric/Fluorometric) (Abcam, ab118970). Intracellular ROS and RNS content was determined using the OxiSelect In Vitro ROS/RNS Assay Kit (Cell Biolabs, STA-324). Extracellular release of ROS (H_2_O_2_) was determined using the Amplex Red Hydrogen Peroxide/Peroxidase Assay Kit (Life Technologies, A22188). All assays were performed following manufacturer’s instructions.

### Reporting summary

Further information on research design is available in the Nature Portfolio Reporting Summary linked to this article.

## Supplementary information

Supplementary information Supplementary Tables 11–16.

Reporting Summary

Supplementary Tables This file contains Supplementary Tables 1–10 along with an information page.

## Data Availability

The murine raw sequencing data have been deposited in the National Center for Biotechnology Information (NCBI) Gene Expression Omnibus (GEO) under accession code GSE230440. Due to the potential risk of de-identification of pseudonymized RNA sequencing data from humans, human raw sequencing data are available under controlled access and require a Data Transfer Agreement in the European Genome-Phenome Archive (EGA) repository, under accession code EGAD00001010301. The human liver dataset can also be explored using our interactive website: https://aouadilabdatabase.org/human_liver_npcs. scRNA-seq data integration with human embryonic liver cells was done on the published dataset of embryonic livers (accession code E-MTAB-7407). Comparison of sequenced cell types from freshly isolated and cryopreserved cells was performed on the published scRNA-seq dataset of human liver cells (accession code GSE124395). The expression of macrophage and dendritic cell markers was compared with a published scRNA-seq dataset of human liver myeloid cells (accession code GSE192742). Flow cytometry data in Extended Data Fig. 7d–f were analyzed from the previously published flow cytometry dataset of human HLA-mismatched liver allografts generated by ref. ^25^. Source data are provided with this paper.

## Source data

Source Data Fig. 1 Raw Giemsa-stained cytospin images as shown in Fig. 1f.
Source Data Fig. 2 Statistical source data.
Source Data Fig. 3 Statistical source data.
Source Data Fig. 4 Statistical source data.
Source Data Fig. 5 Statistical source data.
Source Data Extended Data Fig./Table 1 Statistical source data.
Source Data Extended Data Fig./Table 2 Statistical source data.
Source Data Extended Data Fig./Table 4 Statistical source data.
Source Data Extended Data Fig./Table 6 Statistical source data.
Source Data Extended Data Fig./Table 7 Statistical source data.
Source Data Extended Data Fig./Table 9 Statistical source data.
Source Data Extended Data Fig./Table 10 Statistical source data.
Source Data Extended Data Fig./Table 4 Raw Giemsa-stained cytospin images as shown in Extended Data Fig. 4j.

## Code availability

Bioinformatic analyses and plotting of the scRNA-seq data were performed using Python (v3.6.7) and R software (v4.1.2). All custom scripts can be found at https://github.com/pingchen-angela/liver_scRNAseq.
