# Supplementary material for: Human resident liver myeloid cells protect against metabolic stress in obesity
Source: Nat Metab. 2023 Jul 6;5(7):1188–203. doi: 10.1038/s42255-023-00834-7 (PMC10365994; doi:10.1038/s42255-023-00834-7)
Supplement: Supplementary file 1 — Supplementary Tables 11–16. [file 42255_2023_834_MOESM1_ESM.pdf]

nature metabolism

Article

https://doi.org/10.1038/s42255-023-00834-7

Human resident liver myeloid cells protect against metabolic stress in obesity

In the format provided by the authors and unedited

Supplementary Table 11. Clinical characteristics of patients with obesity included in the study.

Clinical parameters	Obesity and IS (n=4)	Obesity and IR (n=6)	Obesity and T2D (n=3)
Age, years (mean)	43 (29-53)	35 (28-45)	46 (35-56)
Sex, F/M (n)	4/0	6/0	3/0
BMI, kg/m^2, (mean, range)	38 (36-41)	40 (38-41)	36 (34-37)
Diabetes (n)	0	0	3
HSI (mean, range)	51 (49-52)	54 (50-60)	48 (44-50)
Hba1c, mmol/mol (mean, range)	34 (32-37)	36 (27-40)	52 (47-57)
HOMA-IR	2.8 (2.5-3.3)	8.4 (3.7-20.3)	6.1 (5.5-6.8)
Fasting insulin, mIU/L (mean, range)	11 (10-12)	33 (18-75)	17 (13-23)
Fasting Glucose, mmol/L (mean, range)	5.7 (5.2-6.1)	5.4 (4.6-6.1)	8.2 (6.7-9.6)
Creatinine, µmol/L (mean, range)	63 (55-72)	62 (45-70)	75 (58-89)
ALT, µkat/L (mean, range)	0.5 (0.4-0.9)	0.7 (0.3-1.4)	0.5 (0.3-0.6)
AST, µkat/L (mean, range)	0.4 (0.3-0.6)	0.4 (0.2-0.8)	0.5 (0.4-0.5)
GGT, µkat/L (mean, range)	0.7 (0.2-1.1)	1.6 (0.5-2.0)	1.0 (0.8-1.4)
Total Cholesterol, mmol/L (mean, range)	5.1 (3.9-6.2)	5.3 (4.0-6.4)	4.9 (3.7-6.0)
LDL-Cholesterol, mmol/L (mean, range)	3.1 (1.7-4.0)	3.3 (1.8-4.3)	2.7 (1.9-3.3)
HDL-Cholesterol, mmol/L (mean, range)	1.4 (1.2-1.5)	1.1 (1.0-1.3)	1.2 (1.1-1.3)
Triglycerides, mmol/mol (mean, range)	1.8 (0.7-3.1)	2.2 (1.5-3.3)	2.0 (1.2-3.4)

IS, insulin sensitivity; IR, insulin resistance; T2D, type 2 Diabetes; F/M, female/male; BMI, body mass index; HSI, hepatic steatosis index; Hba1c, haemoglobin A1C; HOMA-IR, homeostatic model assessment of insulin resistance; IU, international units; ALT, Alanine aminotransferase; AST, Aspartate aminotransferase; GGT, gamma glutamyl transferase.

Supplementary Table 12. Clinical characteristics of the primary human hepatocytes used in the study.

**Clinical parameters**	Donor 1	Donor 2	Donor 3	Donor 4
Age, years	27	63	50	26
Sex, F/M	F	M	M	F
BMI, kg/m^2	28.2	26.0	20.4	17.6
Pathology	Anoxia	Anoxia	Cerebrovascular accident	Anoxia
Origin	African American	Caucasian	Caucasian	Caucasian
Lipid peroxidation levels (MDA) in healthy conditions (mean, s.e.m.)*	N.A.	2.8 (0.47)	8.4 (0.79)	20.8 (2.33)
Lipid peroxidation levels (MDA) after steatosis induction (mean, s.e.m.)**	N.A.	7.7 (1.18)	24.1 (2.70)	32.2 (1.28)

F/M, female/male; BMI, body mass index; ROS, reactive oxygen species; MDA, malondialdehyde; N.A., not applicable.

*Average peroxidation levels after 48 hours of treatment with healthy medium (0 µM FFA, 5.5 mM glucose, 0.1 nM insulin).

** Average peroxidation levels after 48 hours of treatment with steatogenic medium (480 µM FFA, 11.1 mM glucose, 1.7 µM insulin).

Extended Data Table 13. Antibodies used for flow cytometry.

Antigen	Specie used on	Fluorophore	Clone	Source	Dilution used
**Extracellular staining's**					
Cd11b	Mouse	BB515	M1/70 (RUO)	BD Biosciences	1:100
F4/80	Mouse	APC	CI:A3-1	BioRad	1:10
CD31	Mouse	APC-R700	MEC 13.3	BD Biosciences	1:50
MHCII	Mouse	APC-fire750	M5/114.15.2	Biolegend	1:20
CD49b	Mouse	BV421	HMα2	BD Biosciences	1:20
Cd64	Mouse	BV711	X54-5/7.1	Biolegend	1:20
CD206	Mouse	BV785	C068C2	Biolegend	1:20
ESAM	Mouse	PE	1G8	Thermofisher Scientific	1:50
CD45	Mouse	PE-CF594	30-F11	BD Biosciences	1:100
CD19	Mouse	PE-Cy5	6D5	Biolegend	1:50
CD3e	Mouse	PE-Cy5	145-2C11	BD Biosciences	1:50
Tim4	Mouse	PE-Cy7	RMT4-54	Biolegend	1:50
CD45	Human	AF700	HI30	Biolegend	1:200/1:300
CD45	Human	FITC	HI30	BioLegend	1:100
CD45	Human	BV605	HI30	Biolegend	1:100
CD163	Human	AF647	GHI/61	BD Biosciences	1:50/1:100
CD163	Human	PE	GHI/61	BD Biosciences	1:10
CD8a	Human	APC-Cy7	SK1	BD Biosciences	1:50
BDCA-2	Human	BV421	201A	Biolegend	1:200
CD161	Human	BV650	Dx12	BD Biosciences	1:25
CD3	Human	BV786	SK7	BD Biosciences	1:50
CD3	Human	BB515	UCHT1	BD Biosciences	1:50
CD3	Human	PB	HIT3a	Biolegend	1:50
CD3	Human	BV570	UCHT1	Biolegend	1:100
CD25	Human	BV711	BC96	Biolegend	1:50
CD141	Human	PE	M80	Biolegend	1:100
CD56	Human	PE-CF594	NCAM16.2	BD Bioscience	1:100
CD56	Human	PE-Cy7	NCAM16.2	BD Biosciences	1:50/1:100

CD11c	Human	BV650	B-ly6	BD Biosciences	1:50
CD127	Human	PE-Cy7	A019D5	Biolegend	1:25
CD19	Human	PE-Cy5	HIB19	Biolegend	1:100/1:50
CD19	Human	PB	SJ25C1	Biolegend	1:50
CD19	Human	BV510	SJ25C1	BD Biosciences	1:100
CD14	Human	BV605	M5E2	Biolegend	1:200
CD14	Human	Alexa647	MφP9	BD Biosciences	1:100
CD14	Human	PE-Cy5	61D3	E-bioscience	1:100
TCR Va72	Human	PE	3C10	Biolegend	1:50
CD4	Human	BV570	RPA-T4	Biolegend	1:50
CD206	Human	BB515	19.2	BD Bioscience	1:200
CD206	Human	PE-CF594	19.2	BD Biosciences	1:50
CD206	Human	BV421	19.2	BD Biosciences	1:50
CD206	Human	BB515	19.2	BD Biosciences	1:100
HLA-DR	Human	BUV395	G46-6	BD Bioscience	1:25
HLA-DR	Human	BV785	L243	Biolegend	1:50
HLA-DR	Human	BV711	L243	Biolegend	1:25
HLA-DR	Human	APC-Cy7	L243	Biolegend	1:25/1:50
CD16	Human	BUV737	3G8	BD Bioscience	1:50
CD16	Human	BV786	3G8	BD Biosciences	1:50/1:25
CD16	Human	BV711	3G8	BD Biosciences	1:100
CD31	Human	BV605	WM59	BD Biosciences	1:50
HLA-A3	Human	FITC	REA 950	Miltenyi	1:50
HLA-B5 (51,52)	Human	Biotin	BIH0209	One Lambda/Thermofisher	1:10
HLA-B12	Human	Biotin	REA 138	Miltenyi	1:20
HLA-A11	Human	Biotin	BIH0084	One Lambda/Thermofisher	1:20
HLA-B15, B57	Human	Biotin	BIH0507	One Lambda/Thermofisher	1:10
**Secondary extracellular staining's**					
Streptavidin	N.A.	BV650	nd	BD Bioscience	
**Cell viability staining's**					

Live dead fixable	Aqua	N.A.	N.A.	Invitrogen/Thermofisher	1:1000
Live dead fixable	Violet	N.A.	N.A.	Biolegend	1:1000
Sytox Blue	Blue	N.A.	N.A.	Invitrogen/Thermofisher	1:1000 or 1:3000
**Intracellular staining's**					
CD68	Human	PE-Cy7	Y1/82A	Biolegend	1:16
MRP-14 (S100A9)	Human	PE	MRP 1H9	Biolegend	1:800/1:50

Supplementary Table 14. Antibodies used for Immunofluorescence microscopy.

Target	Clone	Specie	Isotype	Source	Dilution
CD68	KP1 + C68/684	Mouse	IgG1	Abcam	1:50
S100A9	polyclonal	Rabbit	IgG	Abcam	1:50
Ki-67	1O15	Rabbit	IgG	Sigma-Aldrich	1:50
VISIG4	EPR22576-70	Rabbit	IgG	Abcam	1:50
**Secondary staining's**					
Anti-mouse AF647	N.A.	Goat	N.A.	Thermofisher Scientific	1:500
Anti-mouse AF555	N.A.	Goat	N.A.	Thermofisher Scientific	1:500
Anti-rabbit AF488	N.A.	Goat	N.A.	Thermofisher Scientific	1:500
Anti-rabbit AF555	N.A.	Goat	N.A.	Thermofisher Scientific	1:500

Supplementary Table 15. Antibodies used for Phenocycler imaging.

Target	Antibody barcode	Clone	Fluorophore barcode	Source (primary antibody)
CD3e	BX045	EP449E	RX045-Cy5	Abcam
CD11c	BX024	118/A5	RX024-Cy5	Thermofisher
CD31	BX001	EP3095	RX001-AF750	Abcam
CD45RO	BX017	UCHL1	RX017-Atto550	Biolegend
CD68	BX015	KP1	RX015-Cy5	Thermofisher
CD107a	BX006	H4A3	RX006-Cy5	Biolegend
HLA-DR	BX033	EPR3692	RX033-Cy5	Abcam
Ki67	BX047	B56	RX047-Atto550	BD Biosciences
Mac2/Galectin-3	BX035	M3/38	RX035-Atto550	Biolegend
Pan-Cytokeratin	BX019	AE-1/AE-3	RX019- AF750	Biolegend
IDO1	BX027	V1NC3IDO	RX027- CY5	Thermofisher
**In-house conjugated antibodies**				
SMA (ACTA2)	BX028	N.A.	RX028-AF750	N.A.
CD163	BX020	N.A.	RX020-Atto550	N.A.
S100A9	BX021	polyclonal	RX021-Cy5	Abcam

Supplementary Table 16. Phenocycler panel and experimental information.

Phenocycler panel

Cycle	Atto550 (c-2)	Cy5 (c-3)	AF750 (c-4)
Blank	-	-	-
1	Mac2		CD31
2	CD45RO	CD3e	
3		IDO1	SMA
4		CD11c	
5	Ki67	CD107a	
6		HLA-DR	Pan CK
7		CD68	
8	CD163		
9		S100A9	
Blank	-	-	-
