# Supplementary material for: Human resident liver myeloid cells protect against metabolic stress in obesity
Source: Nat Metab. 2023 Jul 6;5(7):1188–203. doi: 10.1038/s42255-023-00834-7 (PMC10365994; doi:10.1038/s42255-023-00834-7)
Supplement: Supplementary file 2 — Reporting Summary [file 42255_2023_834_MOESM2_ESM.pdf]

nature portfolio

Corresponding author(s): Ping Chen and Myriam Aouadi

Last updated by author(s): May 1, 2023

Reporting Summary

Nature Portfolio wishes to improve the reproducibility of the work that we publish. This form provides structure for consistency and transparency in reporting. For further information on Nature Portfolio policies, see our Editorial Policies and the Editorial Policy Checklist.

Statistics

For all statistical analyses, confirm that the following items are present in the figure legend, table legend, main text, or Methods section.

n/a	Confirmed	
☐	☒	The exact sample size (*n*) for each experimental group/condition, given as a discrete number and unit of measurement
☐	☒	A statement on whether measurements were taken from distinct samples or whether the same sample was measured repeatedly
☐	☒	The statistical test(s) used AND whether they are one- or two-sided<br>*Only common tests should be described solely by name; describe more complex techniques in the Methods section.*
☐	☒	A description of all covariates tested
☐	☒	A description of any assumptions or corrections, such as tests of normality and adjustment for multiple comparisons
☐	☒	A full description of the statistical parameters including central tendency (e.g. means) or other basic estimates (e.g. regression coefficient) AND variation (e.g. standard deviation) or associated estimates of uncertainty (e.g. confidence intervals)
☐	☒	For null hypothesis testing, the test statistic (e.g. *F*, *t*, *r*) with confidence intervals, effect sizes, degrees of freedom and *P* value noted<br>*Give P values as exact values whenever suitable.*
☒	☐	For Bayesian analysis, information on the choice of priors and Markov chain Monte Carlo settings
☒	☐	For hierarchical and complex designs, identification of the appropriate level for tests and full reporting of outcomes
☒	☐	Estimates of effect sizes (e.g. Cohen's *d*, Pearson's *r*), indicating how they were calculated

Our web collection on statistics for biologists contains articles on many of the points above.

Software and code

Policy information about availability of computer code

Data collection	All the data are stored on academic servers.<br>Single cell RNA sequencing was performed using a HiSeq 3000 Sequencing System (Illumina).<br>Phenotyping of human tissue and blood samples using flow cytometry was performed on a BD LSR Fortessa equipped with 355 nm, 405 nm, 488 nm, 561 nm and 640 nm lasers, and phenotyping of murine tissues was performed on a BD Symphony A3 equipped with 355 nm, 405 nm, 488 nm, 561 nm and 637 nm lasers.<br>Fluorescence microscopy images used for quantifications were acquired using an inverted Axio Observer Z1 fluorescence microscope (Zeiss) equipped with an Axiocam 506 mono camera (Zeiss). Fluorescence microscopy images used for visualization purposes were acquired either with a Zeiss LSM 880 confocal microscope or a Nikon A1R confocal microscope (Nikon Eclipse Ti).<br>Tissue slides stained with Hematoxylin-eosin and Oil red O staining were scanned with a Panoramic 250 slide scanner.<br>Cytospins stained with Wright-Giemsa were imaged using a Leica DM4000 B microscope.<br>Spatial proteomics was performed using the Akoya Phenocycler-Fusion system (Akoya Biosciences).<br>PRDX2 ELISA, MDA and ROS measurements were performed using a xMark Microplate Absorbance Spectrophotometer (BioRad).<br>Total triglyceride (TG) content and protein concentrations was determined using a SpectraMax i3 Multi-mode microplate reader (Molecular Devices).
Data analysis	Statistical significance of differences between groups was analyzed when appropriate by one-way or two-way analysis of variance (ANOVA) with appropriate post hoc tests, or by two-tailed Mann-Whitney tests using GraphPad Prism Software (version 9.5.0). Data is presented as mean ± s.e.m. p values <0.05 were considered statistically significant.<br>ImageJ2 software (version v1.52h and 2.9.0) was used for quantification and visualization of immunofluorescent images.<br>MATLAB (Version R2022b), QuPath (version 0.3.1) and CytoMAP (version 1.4.21) were used to analyze the spatial proteomics data.<br>QuPath (version 0.3.1) was used to process and visualize the images obtained from Hematoxylin-eosin and Oil red O stainings (tissues) and

Wrigth-Giemsa stainings (cytospins).
FlowJo (version 9.9.6, 10.5.3 and 10.8.1) was used to analyze flow cytometric data and FACSDiva (version 8.0.1., 8.0.2., 9.1.) and Sony Sonia Cell Sorter Software (version 3.1.1.) was used to record flow cytometric data.
The raw reads from single-cell RNA-seq were aligned to the human genome hg38 using STAR aligner (v2.4.2), followed by gene expression quantification using rpkmforgenes. Downstream data analysis and visualization were conducted using Python (version 3.6.7) and R (version 4.1.2) programming languages. Integration of single-cell RNA-seq data from different cohorts was performed using Seurat package (version 4.0.5). RNA velocity and pseudotime analyses were conducted using Velocyto (version 0.17), scVelo (version 0.2.4) and Scanpy (version 1.7.2) Python packages. Comparison of murine and human gene expression were conducted using a list of homologous genes from the Ensembl database (v92) using BioMart.

All custom scripts can be found at https://github.com/pingchen-angela/liver_scRNAseq.

For manuscripts utilizing custom algorithms or software that are central to the research but not yet described in published literature, software must be made available to editors and reviewers. We strongly encourage code deposition in a community repository (e.g. GitHub). See the Nature Portfolio guidelines for submitting code & software for further information.

Data

Policy information about availability of data

All manuscripts must include a data availability statement. This statement should provide the following information, where applicable:

- Accession codes, unique identifiers, or web links for publicly available datasets
- A description of any restrictions on data availability
- For clinical datasets or third party data, please ensure that the statement adheres to our policy

The murine raw sequencing data have been deposited in the NCBI Gene Expression Omnibus (GEO) under accession GSE230440. Due to the potential risk of de-identification of pseudonymized RNA sequencing data from humans, human raw sequencing data are available under controlled access and require a Data Transfer Agreement in the European Genome-Phenome Archive (EGA) repository, under accession EGAD00001010301. The human liver dataset can also be explored using our interactive website: https://aouadilabdatabase.org/human_liver_npcs/. scRNA-seq data integration with human embryonic liver cells was done on the published data set of embryonic livers (accession code: E-MTAB-7407). Comparison of sequenced cell types from freshly isolated and cryopreserved cells was performed on the published scRNA-seq data set of human liver cells (accession code: GSE124395). The expression of macrophage and dendric cell markers was compared to a published scRNA-seq data set of human liver myeloid cells (accession code: GSE192742). Flow cytometry data in Extended Data Fig 7d-7f was analysed from the previously published flow cytometry dataset of human HLA-mismatched liver allografts generated by Pallett et al (25).

Research involving human participants, their data, or biological material

Policy information about studies with human participants or human data. See also policy information about sex, gender (identity/presentation), and sexual orientation and race, ethnicity and racism.

Reporting on sex and gender	Sex was not considered in the study design due to limitations on the number of samples. The number of male and female individuals included in this study have been reported in Table 1-3 and Extended Data Table 1-2.
Reporting on race, ethnicity, or other socially relevant groupings	Not applicable.
Population characteristics	Obese patients: liver samples were obtained from 13 volunteers, with body mass index (BMI) between 35 and 42 (kg/m2), undergoing laparoscopic Roux-en-Y gastric bypass surgery. None of the participants had any previous history of cardiovascular diseases, gastrointestinal diseases, systemic illness, alcohol abuse, coagulopathy, chronic inflammatory diseases, any clinical sign of liver damage or surgical intervention within six months prior to the studies. Patients did not follow any special diet before the surgery. Detailed clinical characteristics are described in Table 1.<br><br>Lean patients: liver samples were obtained from 12 volunteers, with BMI of maximum 25 (kg/m2), undergoing liver resection surgery or collected from donor livers rejected for transplantation. Only non-affected tissues were used.<br><br>For Immunofluorescence: liver samples were obtained from 11 volunteers, with either BMI between 30-38 (5 individuals) or BMI of maximum 25 (6 individuals), undergoing liver resection surgery.<br><br>For patients undergoing liver transplantation: liver and blood samples were obtained from 3 volunteers undergoing liver transplantation surgery with HLA-mismatched liver allografts. The patients underwent liver transplantation surgery due to either primary sclerosing cholangitis, primary biliary cirrhosis or progressive familial intrahepatic cholestasis.
Recruitment	Patients were recruited by doctors at the Danderyd hospital in Stockholm and at the Karolinska Hospital, Huddinge. A research nurse called the patients for consent and subsequent visits. No compensation or any other form of payment was given to participants, to ensure that samples are donated out of free will and for no other reason. We have not identified any selection bias that would impact the results.
Ethics oversight	All the studies involving human subjects have been planned in compliance with national legislation and the Code of Ethical Principles for Medical Research Involving Human Subjects of the World Medical Association (Declaration of Helsinki), and have been granted ethical approvals by the Regional Ethical Review Board in Sweden (Regional Ethical Committee in Stockholm, Sweden (2017/214-31, 2017/269-31, 2008/1010-31, 2006/229-31) and all patients provided oral and written informed consent.

Note that full information on the approval of the study protocol must also be provided in the manuscript.

Field-specific reporting

Please select the one below that is the best fit for your research. If you are not sure, read the appropriate sections before making your selection.

☒ Life sciences ☐ Behavioural & social sciences ☐ Ecological, evolutionary & environmental sciences

For a reference copy of the document with all sections, see nature.com/documents/nr-reporting-summary-flat.pdf

Life sciences study design

All studies must disclose on these points even when the disclosure is negative.

Sample size	For the single cell RNA sequencing data from human and mice, sample sizes were not predetermined. Sample sizes were based on our experience, common practice in the field, statistical robustness and resource availability. We obtained reproducible differences between conditions indicating that the sample sizes were sufficient.<br>For all the animal experiments and human in vitro experiments, sample sizes were calculated based on previous data collection as described in Rosner, B. Fundamental of Biostatistics 7th edn (Brooks/Cole CENGAGE Learning, 2010).
Data exclusions	For the recruitment of human patients, inclusion/exclusion criteria are stated in the method section. Low quality cells that did not fulfill the quality control criteria, from the single cell RNA sequencing data, were excluded from subsequent analysis.
Replication	Biological replicates were included to ensure the reproducibility and all repeated experiments were successful. For single-cell RNA sequencing experiments, sequencing was performed on 1-2 technical replicates for each patient/sample. Findings identified by single cell transcriptomes were successfully validated using experimental assays including immunostaining, cytospin, flow cytometric analysis and functional in vitro assays were carried out. For all experiments at least three biological replicates were included and experiments were performed independently to ensure reproducibility. For animal experiments, the number of replicates is equal to individual mice used. When representative data are shown, the experimental findings were reproduced independently with similar results.
Randomization	All mice were randomly assigned into control and experimental groups. For ex vivo and in vitro experiments, the samples and treatments were randomized.
Blinding	Investigators were blinded for the Immunofluorescent quantifications performed on human liver tissues. Investigators were not blinded for analyses on measurements of other quantitative parameters as all experiments were unbiased and the conclusions were based on multiple independent experiments and statistical significance.

Reporting for specific materials, systems and methods

We require information from authors about some types of materials, experimental systems and methods used in many studies. Here, indicate whether each material, system or method listed is relevant to your study. If you are not sure if a list item applies to your research, read the appropriate section before selecting a response.

Materials & experimental systems

n/a	Involved in the study	
☐	☒	Antibodies
☒	☐	Eukaryotic cell lines
☒	☐	Palaeontology and archaeology
☐	☒	Animals and other organisms
☒	☐	Clinical data
☒	☐	Dual use research of concern
☒	☐	Plants

Methods

n/a	Involved in the study	
☒	☐	ChIP-seq
☐	☒	Flow cytometry
☒	☐	MRI-based neuroimaging

Antibodies

Antibodies used	Human antibodies used for FACS or flow cytometry (company; clone name; dilution):<br>CD45-FITC (BioLegend; HI30; 1:100), CD206-PE-CF594 (BD Bioscience; 19.2; 1:50), CD16-BV786 (BD Biosciences; 3G8; 1:50/1:25), CD31-BV605 (BD Biosciences; WM59; 1:50), CD3-PB (BioLegend; HIT3a; 1:50), CD19-PB (BioLegend; SJ25C1; 1: 50), HLA-DR-APC-Cy7 (BioLegend; L243; 1:50/1:25), CD14-AF647 (BD Bioscience, MϕP9; 1:100), HLA-DR-BV711 (Biolegend; L243; 1:25), CD3-BB515 (BD Biosciences; UCHT1; 1:50), CD45-AF700 (Biolegend; HI30; 1:300), CD163-AF647 (BD Biosciences; 1:50/1:100), CD8a-APC-Cy7 (BD Biosciences; SK1; 1:50), BDCA-2-BV421 (Biolegend; 201A; 1:200), CD161-BV650 (BD Biosciences; Dx12; 1: 25), CD3-BV786 (BD Biosciences, SK7; 1:50), CD25-BV711 (BioLegend; BC96; 1:50), CD141-PE (Biolegend; M80; 1:100), TCR Va72-PE (Biolegend; 3C10; 1:50), CD56-PE-CF594 (BD Bioscience; NCAM16.2; 1:100), CD127-PE-Cy7 (BioLegend; A019D5; 1:25), CD19-PE-Cy5 (Biolegend; HIB19; 1:100), CD14-BV605 (Biolegend; M5E2; 1:200), CD4-BV570 (BioLegend; RPA-T4; 1:50), HLA-DR (BD Bioscience; G46-6; 1:25), CD16-BUV737 (BD Bioscience; 3G8; 1:50), CD68-PE-Cy7 (Biolegend; Y1/82A; 1: 16), CD19-BV510 (BD Biosciences; SJ25C1; 1: 100), CD14-PE-Cy5 (E-bioscience; 61D3; 1:100), CD206-BV421 (BD Biosciences; 19.2; 1:50), MRP-14 (S100A9)-PE (Biolegend; MRP 1H9; 1:800), CD206-BB515 (BD Bioscience; 19.2; 1:100), CD45-BV605 (Biolegend; HI30 ; 1:100), HLA-DR-BV785 (Biolegend; L243; 1:50), CD16-BV711 (BD Biosciences; 3G8; 1:100), CD56-PE-Cy7 (BD Biosciences; NCAM16.2; 1:100), CD3-BV570 (Biolegend; UCHT1; 1:100).

Murine antibodies used for FACS or Flow cytometry (company; clone name; dilution):
Cd11b-BB515 (BD Biosciences; M1/70(RUO); 1:100), F4/80-APC (BioRad; MCA497APC; 1:10), CD31-APC-R700 (BD Biosciences; MEC 13.3; 1:50), MHCII-APC-fire750 (Biolegend; M5/114.15.2; 1:20), CD49b-BV421 (BD Biosciences; HMα2; 1:20), Cd64-BV711(Biolegend; X54-5/7.1; 1:20), CD206-BV785 (Biolegend; C068C2; 1:20), ESAM-PE (Thermofisher Scientific; 1G8; 1:50), CD45-PE-CF594 (BD Biosciences; 30-F11; 1: 100), CD19-PE-Cy5 (Biolegend; 6D5; 1:50/1:50), CD3e-PE-Cy5 (BD Biosciences; 145-2C11; 1:50), Tim4-PE-Cy7 (Biolegend; RMT4-54; 1:50).

Primary antibodies used for Immunofluorescence (company; clone name, dilution):
CD68 (Abcam; KP1 + C68/684; 1:50), S100A9 (Abcam; polyclonal; 1:50), Ki-67 (Sigma-Aldrich; 1O15; 1:50) and VISIG4 (Abcam; EPR22576-70; 1:50).

Secondary antibodies used for Immunofluorescence (company; dilution):
Goat anti-mouse AF647 (Thermofisher Scientific; 1:500), Goat anti-mouse AF555 (Thermofisher Scientific; 1:500), Goat anti-rabbit AF488 (Thermofisher Scientific; 1:500) and Goat anti-rabbit AF555 (Thermofisher Scientific; 1:500).

Primary antibodies (company; clone name)-barcodes-fluorophores used for Spatial proteomics:
CD3e (Abcam; EP449E)-BX045-Cy5, CD11c (Thermofisher; 118/A5)-BX024-Cy5, CD31 (Abcam; EP3095)-BX001-AF750, CD45RO (Biolegend; UCHL1)-BX017- Atto550, CD68 (Thermofisher; KP1)-BX015-Cy5, CD107a (Biolegend; H4A3)-BX006-Cy5, HLA-DR (Abcam; EPR3692)-BX033-Cy5, Ki67 (BD Biosciences; B56)-BX047-Atto550, Mac2/Galectin-3 (Biolegend; M3/38)-BX035-Atto 550, Pan-Cytokeratin (Biolegend; AE-1/AE-3)-BX019-AF750, IDO1 (Thermofisher; V1NC3IDO)-BX027-CY5, SMA(ACTA2) (N.A.)-BX028-AF750, CD163 (N.A.)-BX020-Atto550, and S100A9 (Abcam; polyclonal)-BX021-Cy5.

Validation

All antibodies were commercially available and validated for the species and application by the company, as well as other researchers (see below). All antibodies were also tested by us and titrated before use in experiments. Fluorescence minus controls (FMOs) were used to validate the stainings of facs antibodies, while stainings with secondary antibody only (withouth primary) was used as negative control for in all stainings with IF.

Human CD45-AF700: https://www.biolegend.com/fr-lu/products/pe-dazzle-594-anti-human-cd1c-antibody-10808
Human CD163-AF647: https://www.bdbiosciences.com/en-se/products/reagents/flow-cytometry-reagents/research-reagents/single-color-antibodies-ruo/alexa-fluor-647-mouse-anti-human-cd163.562669
Human CD8a-APCCy7: https://www.bdbiosciences.com/en-se/products/reagents/flow-cytometry-reagents/research-reagents/single-color-antibodies-ruo/apc-cy-7-mouse-anti-human-cd8.557834
Human BDCA-2-BV421: https://www.biolegend.com/fr-lu/products/brilliant-violet-421-anti-human-cd303-bdca-2-antibody-8709
Human CD161-BV650: https://www.bdbiosciences.com/en-se/products/reagents/flow-cytometry-reagents/research-reagents/single-color-antibodies-ruo/bv650-mouse-anti-human-cd161.563864
Human CD3-BV786: https://www.bdbiosciences.com/en-se/products/reagents/flow-cytometry-reagents/research-reagents/single-color-antibodies-ruo/bv786-mouse-anti-human-cd3.563800
Human CD25-BV711: https://www.biolegend.com/en-us/products/brilliant-violet-711-anti-human-cd25-antibody-7934
Human CD141-PE: https://www.biolegend.com/en-us/products/pe-anti-human-cd141-thrombomodulin-antibody-6107
Human TCRVa72-PE: https://www.biolegend.com/en-us/products/pe-anti-human-tcr-valpha7-2-antibody-7124
Human CD56-PECF594: https://www.bdbiosciences.com/en-se/products/reagents/flow-cytometry-reagents/research-reagents/single-color-antibodies-ruo/pe-cf594-mouse-anti-human-cd56.564849
Human CD127-PECy7: https://www.biolegend.com/en-us/products/pe-cyanine7-anti-human-cd127-il-7ralpha-antibody-7216
Human CD19-PECy5: https://www.biolegend.com/en-us/products/pe-cyanine5-anti-human-cd19-antibody-720
Human CD14-BV605: https://www.biolegend.com/en-us/products/brilliant-violet-605-anti-human-cd14-antibody-7653
Human CD4-BV570: https://www.biolegend.com/en-us/products/brilliant-violet-570-anti-human-cd4-antibody-7359
Human HLA-DR-BUV395: https://www.bdbiosciences.com/en-se/products/reagents/flow-cytometry-reagents/research-reagents/single-color-antibodies-ruo/buv395-mouse-anti-human-hla-dr.564040
Human CD16-BUV737: https://www.bdbiosciences.com/en-se/products/reagents/flow-cytometry-reagents/research-reagents/single-color-antibodies-ruo/buv737-mouse-anti-human-cd16.612786
Human CD68-PE-Cy7: https://www.biolegend.com/en-us/products/pe-cyanine7-anti-human-cd68-antibody-9123
Human CD19-BV510: https://www.bdbiosciences.com/en-se/products/reagents/flow-cytometry-reagents/research-reagents/single-color-antibodies-ruo/bv510-mouse-anti-human-cd19.562947
Human CD14-PE-Cy5: https://www.thermofisher.com/antibody/product/CD14-Antibody-clone-61D3-Monoclonal/15-0149-42
Human CD206-BV421: https://www.bdbiosciences.com/en-se/products/reagents/flow-cytometry-reagents/research-reagents/single-color-antibodies-ruo/bv421-mouse-anti-human-cd206.564062
Human MRP-14(S100A9)-PE: https://www.biolegend.com/en-us/products/pe-anti-human-mrp-14-s100a9-antibody-10078
Human CD206-BB515: https://www.bdbiosciences.com/en-se/products/reagents/flow-cytometry-reagents/research-reagents/single-color-antibodies-ruo/bb515-mouse-anti-human-cd206.564668
Human CD45-BV605: https://www.biolegend.com/en-us/products/brilliant-violet-605-anti-human-cd45-antibody-8521
Human HLA-DR-BV785: https://www.biolegend.com/en-us/products/brilliant-violet-785-anti-human-hla-dr-antibody-7975
Human CD16-BV711: https://www.biolegend.com/en-us/products/brilliant-violet-785-anti-human-hla-dr-antibody-7975
Human CD56-PECy7: https://www.bdbiosciences.com/en-se/products/reagents/flow-cytometry-reagents/clinical-diagnostics/single-color-antibodies-asr-ivd-ce-ivd/cd56-pe-cy-7.335826
Human CD3-BV570: https://www.biolegend.com/en-us/products/brilliant-violet-570-anti-human-cd3-antibody-7368
Human HLA-A3: https://www.thermofisher.com/onelambda/wo/en/products.html?articleNumber=BIH0209
Human HLA-B5: https://www.thermofisher.com/onelambda/wo/en/products.html?articleNumber=BIH0209
Human HLA-B12: https://www.miltenyibiotec.com/SE-en/products/hla-b12-antibody-anti-human-reafinity-rea138.html#conjugate=vio-bright-r720:size=100-tests-in-200-ul
Human HLA-A11: https://www.thermofisher.com/onelambda/wo/en/products.html?articleNumber=BIH0084
Human HLA-B15, B57: https://www.thermofisher.com/onelambda/wo/en/products.html?articleNumber=BIH0507
Human CD45 FITC: https://www.biolegend.com/en-us/products/fitc-anti-human-cd45-antibody-707
Human CD206-PECF594: https://www.bdbiosciences.com/en-se/products/reagents/flow-cytometry-reagents/research-reagents/single-color-antibodies-ruo/pe-cf594-mouse-anti-human-cd206.564063
Human CD16-BV786: https://www.bdbiosciences.com/en-se/products/reagents/flow-cytometry-reagents/research-reagents/single-

color-antibodies-ruo/bv786-mouse-anti-human-cd16.563690
Human CD31-BV605: https://www.bdbiosciences.com/en-se/products/reagents/flow-cytometry-reagents/research-reagents/single-color-antibodies-ruo/bv605-mouse-anti-human-cd31.562855
Human CD3-PB: https://www.biolegend.com/en-us/products/pacific-blue-anti-human-cd3-antibody-6505
Human CD19-PB: https://www.biolegend.com/en-us/products/pacific-blue-anti-human-cd19-antibody-13773
Human HLA-DR-APCCy7: https://www.biolegend.com/en-us/products/apc-cyanine7-anti-human-hla-dr-antibody-2863
Human CD14-AF647: https://www.bdbiosciences.com/en-se/products/reagents/flow-cytometry-reagents/research-reagents/single-color-antibodies-ruo/alexa-fluor-647-mouse-anti-human-cd14.562690
Human HLA-DR-BV711: https://www.biolegend.com/en-us/products/brilliant-violet-711-anti-human-hla-dr-antibody-7939
Human CD3-BB515: https://www.bdbiosciences.com/en-se/products/reagents/flow-cytometry-reagents/research-reagents/single-color-antibodies-ruo/bb515-mouse-anti-human-cd3.564466
Human CD11c-BV650: https://www.bdbiosciences.com/en-se/products/reagents/flow-cytometry-reagents/research-reagents/single-color-antibodies-ruo/bv650-mouse-anti-human-cd11c.563403
Human CD163-PE: https://www.bdbiosciences.com/en-se/products/reagents/flow-cytometry-reagents/research-reagents/single-color-antibodies-ruo/pe-mouse-anti-human-cd163.560933
Mouse CD11b-BB515: https://www.bdbiosciences.com/en-se/products/reagents/flow-cytometry-reagents/research-reagents/single-color-antibodies-ruo/bb515-rat-anti-cd11b.564454
Mouse F4/80-APC: https://www.bio-rad-antibodies.com/monoclonal/mouse-f4-80-antibody-cl-a3-1-mca497.html?f=purified&JSESSIONID_STERLING=76F26DC81B988080BE7D04E87885C01A.ecommerce1&evCntryLang=SE-EN&EU_COOKIE_PREFS=000&cntry=SE&thirdPartyCookieEnabled=true
Mouse CD31-APCR700: https://www.bdbiosciences.com/content/dam/bdb/products/global/reagents/flow-cytometry-reagents/research-reagents/single-color-antibodies-ruo/565509_base/pdf/565509.pdf
Mouse MHCII-APCfire750: https://www.biolegend.com/en-us/products/apc-fire-750-anti-mouse-i-a-i-e-antibody-13215
Mouse CD49b-BV421: https://www.bdbiosciences.com/en-se/products/reagents/flow-cytometry-reagents/research-reagents/single-color-antibodies-ruo/bv421-hamster-anti-mouse-cd49b.740030
Mouse Cd64-BV711: https://www.biolegend.com/en-us/products/brilliant-violet-711-anti-mouse-cd64-fcgammari-antibody-9920
Mouse CD206-BV785: https://www.biolegend.com/en-us/products/brilliant-violet-785-anti-mouse-cd206-mmr-antibody-12013
Mouse ESAM-PE: https://www.thermofisher.com/antibody/product/ESAM-Antibody-clone-1G8-Monoclonal/12-5852-82
Mouse CD45-PECF594: https://www.bdbiosciences.com/en-se/products/reagents/flow-cytometry-reagents/research-reagents/single-color-antibodies-ruo/pe-cf594-rat-anti-mouse-cd45.562420
Mouse CD19-PECy5: https://www.biolegend.com/en-us/products/pe-cyanine5-anti-mouse-cd19-antibody-1531
Mouse CD3e-PE-Cy5: https://www.bdbiosciences.com/en-se/products/reagents/flow-cytometry-reagents/research-reagents/single-color-antibodies-ruo/pe-cy-5-hamster-anti-mouse-cd3e.553065
Mouse Tim4-PECy7: https://www.biolegend.com/en-us/products/pe-cyanine7-anti-mouse-tim-4-antibody-11944

Antibodies used for IF:
CD68: https://www.abcam.com/products/primary-antibodies/cd68-antibody-kp1--c68684-ab199000.html
S100A9: https://www.abcam.com/products/primary-antibodies/s100a9-antibody-ab63818.html
Ki-67: https://www.sigmaaldrich.com/SE/en/product/sigma/zrb1007
VISIG4: https://www.abcam.com/products/primary-antibodies/vsig4-antibody-epr22576-70-ab252933.html

Goat anti-mouse AF647: https://www.thermofisher.com/antibody/product/Goat-anti-Mouse-IgG-H-L-Highly-Cross-Adsorbed-Secondary-Antibody-Polyclonal/A-21236
Goat anti-mouse AF555: https://www.thermofisher.com/antibody/product/Goat-anti-Mouse-IgG-H-L-Cross-Adsorbed-Secondary-Antibody-Polyclonal/A-21422
Goat anti-rabbit AF488: https://www.thermofisher.com/antibody/product/Goat-anti-Rabbit-IgG-H-L-Highly-Cross-Adsorbed-Secondary-Antibody-Polyclonal/A-11034
Goat anti-rabbit AF555: https://www.thermofisher.com/antibody/product/Goat-anti-Rabbit-IgG-H-L-Cross-Adsorbed-Secondary-Antibody-Polyclonal/A-21428

Animals and other research organisms

Policy information about studies involving animals; ARRIVE guidelines recommended for reporting animal research, and Sex and Gender in Research

Laboratory animals	Four-week-old wild type C57BL/6J males were obtained from Charles River Laboratories International, Inc.. Mice were group-housed under specific pathogen-free conditions and maintained on a 12-hour light/dark cycle at 20°C ±1°C with 50-53% humidity with ad libitum access to food and water. Male mice were used in all the experiments due to the predominant use of male mice in previous studies of diet-induced obesity and metabolic disease. Experiments were performed on mice between the age of 5 to 15 weeks. Professor Stephan P. Rosshart (Friedrich-Alexander-University Erlangen-Nürnberg, Erlangen, Germany and University of Freiburg, Freiburg im Breisgau, Germany) kindly provided C57BL/6NTac wildling mice and C57BL/6NTac pathogen free control mice.
Wild animals	The study did not involve wild animals.
Reporting on sex	Male mice were used in all the experiments due to the predominant use of male mice in previous studies of diet-induced obesity and metabolic disease. The sex of the mice was assigned by the technicians at Charles River Laboratories International and checked by the animal facility technicians by genitalia observations.
Field-collected samples	The study did not involve samples collected in the field.
Ethics oversight	All procedures were performed in accordance with guidelines approved by the Regional Ethical Committee in Stockholm (Stockholms djurförsöksetiska nämnd, Stockholms södra djurförsöksetiska nämnd and Linköpings djurförsöksetiska nämnd).

Note that full information on the approval of the study protocol must also be provided in the manuscript.

Flow Cytometry

Plots

Confirm that:

☒ The axis labels state the marker and fluorochrome used (e.g. CD4-FITC).

☒ The axis scales are clearly visible. Include numbers along axes only for bottom left plot of group (a 'group' is an analysis of identical markers).

☒ All plots are contour plots with outliers or pseudocolor plots.

☒ A numerical value for number of cells or percentage (with statistics) is provided.

Methodology

Sample preparation	Non-parenchymal liver cells from obese individuals were isolated by from liver samples collected during gastric bypass surgery and were processed by mechanical dissociation under sterile conditions. Samples were then digested with 0.25 mg/mL collagenase II and 0.2 mg/mL DNase I in a shaking water bath at 37°C approximately 30 min. The cell suspension was then filtered through a 70µm cell strainer and centrifuged at 50 g for 3 min at 4°C to pellet the hepatocytes. The supernatant containing the NPCs was subsequently washed once in PBS and cryopreserved in FBS or FCS with 10% DMSO and stored in liquid nitrogen until further use.<br><br>Non-parenchymal liver cells from lean individuals were isolated from liver samples collected during liver resection surgery and liver samples were processed through a three-step perfusion technique. In summary, livers excess sinusoidal blood (intrahepatic blood) was collected in a series of flushing steps. Subsequently, livers were subjected to several perfusion steps including enzymatic digestion with Collagenase XI (Sigma). Cells were then cryopreserved in FBS with 10% DMSO and stored in liquid nitrogen until further use.<br><br>Non-parenchymal liver cells from lean and obese C57BL/6 mice were isolated by liver perfusion. Briefly, anesthetized mice were perfused with calcium-free Hanks' balanced salt solution (HBSS), followed by perfusion with collagenase. After digestion, livers were collected, and cells released by mechanical dissociation. The cell suspension was filtered through a 100µm mesh and centrifuged at 50g for 3 min to pellet the hepatocytes. The resulting supernatant containing the NPCs was collected and washed twice with PBS to be used for single cell sorting<br><br>Non-parenchymal liver cells from C57BL/6NTac wildling mice and C57BL/6Ntac control mice were isolated by first euthanising the animals followed by perfusion of the liver with PBS. The perfused livers were collected in PBS and then processed by mechanical dissociation under sterile conditions before digestion with 0.25 mg/mL collagenase II and 0.2 mg/mL DNase I in a shaking water bath at 37°C approximately 30 min until digested. The cell suspension was then filtered through a 70µm cell strainer and centrifuged at 50 g for 3 min at 4°C to pellet the hepatocytes. The supernatant containing the NPCs was subsequently washed twice in PBS.<br><br>PBMCs from blood samples or mononuclear cells from intrahepatic blood were isolated with density gradient centrifugation.
Instrument	Sony MA900 (equipped with 3 lasers), BD FACSAria Fusion (equipped with 4 lasers), BD LSR Fortessa (equipped with 5 lasers) and BD Symphony A3 (equipped with 5 lasers)
Software	FACSDiva (version 8.0.1., 8.0.2., 9.1.), Sony Sonia Cell Sorter Software (version 3.1.1.) and FlowJo (version 9.9.6, 10.5.3 and 10.8.1).
Cell population abundance	The abundance of human myeloid cells in total NPC fraction is ~ 1-3%.<br>Purity was determined by flow cytometry and confirmed in our data analysis.
Gating strategy	For sorting of NPCs from obese individuals for scRNA-seq, dead cell marker/FSC-A gating was used to detect live cells, FSC-W/FSC-H and SSC-W/SSC-H was used to gate out single cells.<br>For sorting of NPCs from lean individuals for scRNA-seq, myeloid cells were enriched by FSC-A/SSC-A gating to detect all cells, dead cell marker/FSC-A gating was used to detect live cells, FSC-W/FSC-A was used to gate out single cells, CD45/CD3 was used to gate on CD45+ and CD3+ cells, CD3/FSC-A was used to exclude CD3+ cells, CD56/CD19 was used to exclude CD56+ and CD19+ cells.<br>For sorting of NPCs from lean and obese mice, FSC-A/SSC-A gating was used to detect all events, FSC-H/FSC-A and SSC-H/SSC-A gating was used to detect single cells and dead cell marker/FSC-A gating was used to select live cells.<br>For Sorting of human LM1-LM4, FSC-A/SSC-A gating was done to detect all cells, FSC-H/FSC-A gating was used to detect single cells and dead cell marker, CD3,CD19/FSC-A gating was used to select live CD3-CD19- cells, CD45/FSC-A gating was done to select CD45+ cells, CD14/HLA-DR gating was done to select CD14+HLA-DR+ cells, CD16/CD206 gating was done to sort LM1-LM4 cells.<br>For flow cytometric analyses, FSC-A/SSC-A was either used to detect and analyze single cells or FSC-H/FSC-A was used to detect and analyze single cells. Further gating depends on the experiment strategy as described in the manuscript and associated extended data.

☒ Tick this box to confirm that a figure exemplifying the gating strategy is provided in the Supplementary Information.
