# Supplementary figures and images for: Human resident liver myeloid cells protect against metabolic stress in obesity
Source: Nat Metab. 2023 Jul 6;5(7):1188–203. doi: 10.1038/s42255-023-00834-7 (PMC10365994; doi:10.1038/s42255-023-00834-7)

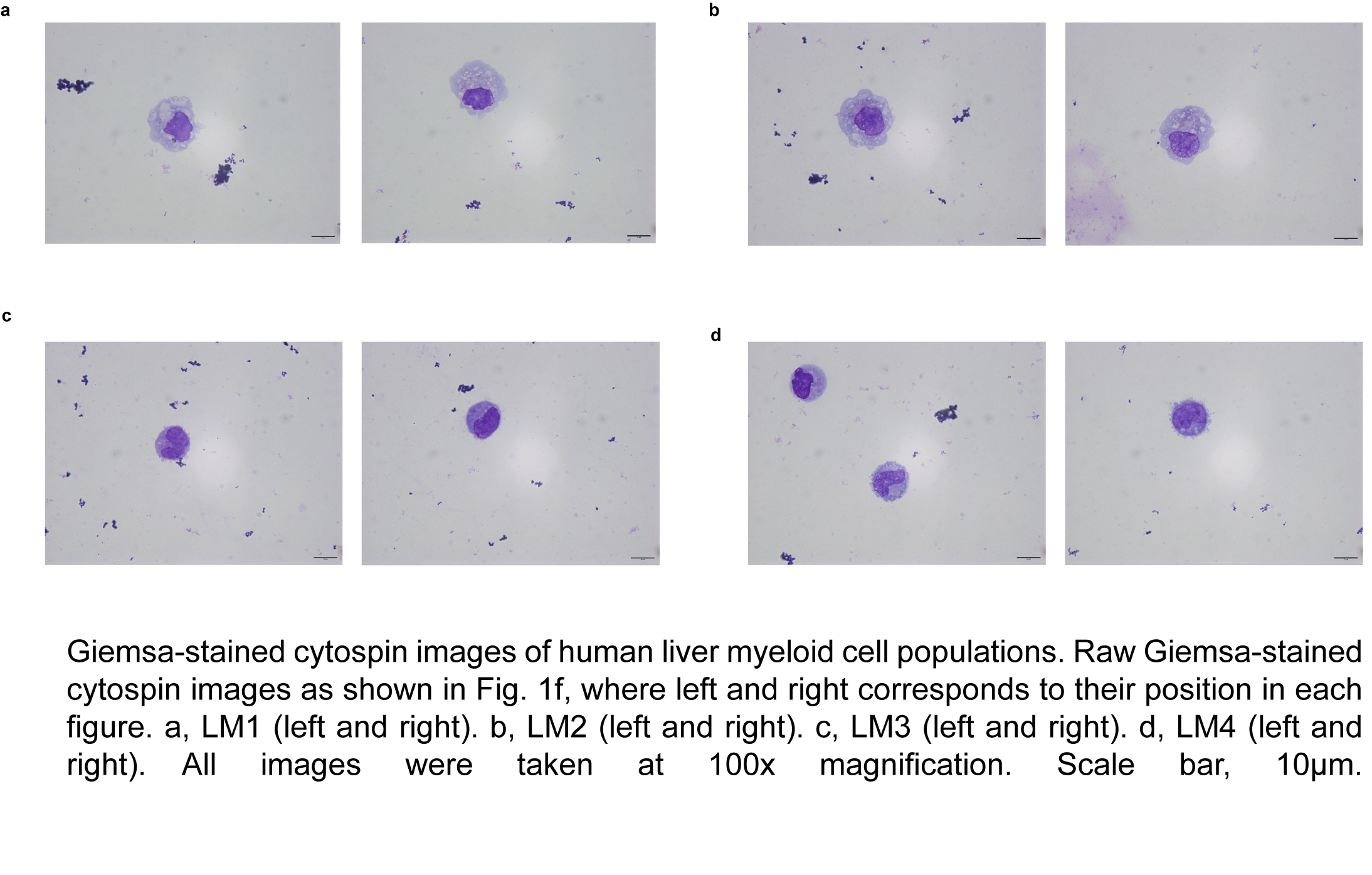

Supplement: Source Data Fig. 1 — Raw Giemsa-stained cytospin images as shown in Fig. 1f. [file 42255_2023_834_MOESM4_ESM.tif]

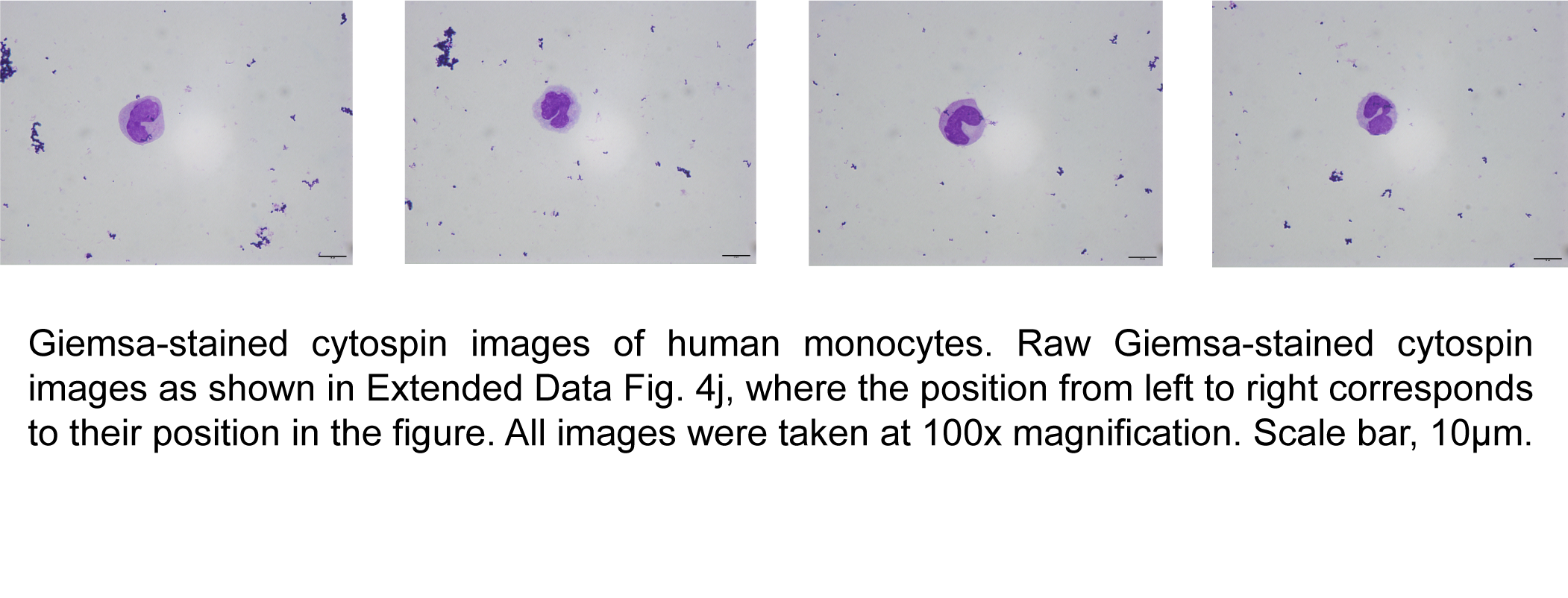

Supplement: Source Data Extended Data Fig./Table 4 — Raw Giemsa-stained cytospin images as shown in Extended Data Fig. 4j. [file 42255_2023_834_MOESM16_ESM.tif]
